# Whole genome duplication and transposable element proliferation drive genome expansion in Corydoradinae catfishes

**DOI:** 10.1098/rspb.2017.2732

**Published:** 2018-02-14

**Authors:** Sarah Marburger, Markos A. Alexandrou, John B. Taggart, Simon Creer, Gary Carvalho, Claudio Oliveira, Martin I. Taylor

**Affiliations:** 1 Institute of Aquaculture, University of Stirling, Stirling FK9 4LA, UK; 2 Molecular Ecology and Fisheries Genetics Laboratory, School of Biological Sciences, Bangor University, Deiniol Road, Bangor, Gwynedd LL57 2UW, UK; 3 Departamento de Morfologia, Instituto de Biociências/UNESP, Rua Professor Doutor Antonio Celso Wagner Zanin, s/n°18618-689 Botucatu, São Paulo, Brazil; 4 School of Biological Sciences, University of East Anglia, Norwich NR4 7TJ, UK; 5 Wildlands Conservation Science, LLC PO Box 1846, Lompoc, CA 93438, USA

**Keywords:** genome size evolution, WGD, polyploidy, Corydoras, transposable elements

## Abstract

Genome size varies significantly across eukaryotic taxa and the largest changes are typically driven by macro-mutations such as whole genome duplications (WGDs) and proliferation of repetitive elements. These two processes may affect the evolutionary potential of lineages by increasing genetic variation and changing gene expression. Here, we elucidate the evolutionary history and mechanisms underpinning genome size variation in a species-rich group of Neotropical catfishes (Corydoradinae) with extreme variation in genome size—0.6 to 4.4 pg per haploid cell. First, genome size was quantified in 65 species and mapped onto a novel fossil-calibrated phylogeny. Two evolutionary shifts in genome size were identified across the tree—the first between 43 and 49 Ma (95% highest posterior density (HPD) 36.2–68.1 Ma) and the second at approximately 19 Ma (95% HPD 15.3–30.14 Ma). Second, restriction-site-associated DNA (RAD) sequencing was used to identify potential WGD events and quantify transposable element (TE) abundance in different lineages. Evidence of two lineage-scale WGDs was identified across the phylogeny, the first event occurring between 54 and 66 Ma (95% HPD 42.56–99.5 Ma) and the second at 20–30 Ma (95% HPD 15.3–45 Ma) based on haplotype numbers per contig and between 35 and 44 Ma (95% HPD 30.29–64.51 Ma) and 20–30 Ma (95% HPD 15.3–45 Ma) based on SNP read ratios. TE abundance increased considerably in parallel with genome size, with a single TE-family (TC1-IS630-Pogo) showing several increases across the Corydoradinae, with the most recent at 20–30 Ma (95% HPD 15.3–45 Ma) and an older event at 35–44 Ma (95% HPD 30.29–64.51 Ma). We identified signals congruent with two WGD duplication events, as well as an increase in TE abundance across different lineages, making the Corydoradinae an excellent model system to study the effects of WGD and TEs on genome and organismal evolution.

## 1. Introduction

There is spectacular variation in genome size across the animal and plant kingdoms, with 200 000-fold variation reported across the eukaryotes [1]. However, the long-term evolutionary consequences of such variation in genome size among taxa remain poorly understood. Genome size affects some key physiological traits such as cell size [1] and metabolic rate [2], though ‘organismal complexity’ and the number of genes in an organism's genome are not necessarily related to genome size [1]. Increases in genome size may be driven by several processes, including whole genome duplications (WGDs), transposable element (TE) proliferation, intron expansion and tandem gene duplications [3]. Of these, arguably the most significant in terms of the speed and scale of genome size change are WGDs and TE proliferation [3].

WGDs have played important roles in both the mode and tempo of evolution in a variety of organisms [4]. They are particularly common in plants and have been implicated in their evolutionary success [4]. Multiple rounds of WGD have also occurred in the vertebrate lineage with an additional genome duplication having occurred in the common teleost ancestor [5], with further duplications having occurred in some teleost lineages including the salmonids [6]. WGD can lead to profound genomic changes, including the retention of duplicated genes with potential to evolve novel functions [7], accumulation of TEs [6,8] increases in the diversity of miRNA family members [9] and the rearrangement of chromosomes [10].

The accumulation of repetitive elements and TE expansions can also lead to rapid increases in genome size and this may be independent of, or in concert with, WGD [11]. Maize is one of the most dramatic examples of post-WGD TE expansion where 85% of the genome is composed of TEs [12]. While TE insertions are generally considered deleterious [13], TEs may also play a role in adaptation. For example, TE insertions have been linked with insecticide resistance in *Drosophila* [14], with increased diversity and adaptive genomic islands in an invasive ant [15] and melanism mutation in peppered moths (*Biston betularia*) [16].

Here, we focus on the Neotropical Corydoradinae catfishes, which are a species-rich group comprising some 170 described species with many further undescribed taxa [17]. Variation in genome size among species is high, with C-values ranging from 0.6 pg to more than 4 pg with *Corydoras aeneus* having the largest currently recorded genome of any teleost fish at 4.4 pg (http://www.genomesize.com/). Diploid karyotypes range from 46 to 134 chromosomes [18], with evidence of extensive chromosomal fusions in high genome size species [19]. Despite decades of interest in the group with regard to genome size and chromosomal diversity, the origins and tempo of genome size change within the group have remained enigmatic. Understanding has been impeded by the lack of a robust phylogenetic framework, the high taxon diversity and the occurrence of colour pattern mimicry complicating species identification [20]. However, recent phylogenetic analysis of the group has established a comprehensive molecular mtDNA phylogeny [20] facilitating more detailed investigation of the evolution of genome size within the group. The multiple lineages identified and the comparison between diploid and potentially polyploid lineages makes the Corydoradinae an interesting and powerful model system to study the evolutionary implications of WGD and TE proliferation.

In this study, we investigate the evolutionary history of genome size change within the Corydoradinae and investigate two mechanisms that may underpin genome size expansion: WGD and repetitive element proliferation. To this end, we (i) constructed a comprehensive fossil-calibrated molecular phylogeny using an uncorrelated relaxed clock which provides a framework for dating genome size changes, (ii) estimated haploid nuclear DNA content (referred to as the C-value throughout) for representatives of all known Corydoradinae lineages using Feulgen Image Densitometry, (iii) employed restriction-site-associated DNA (RAD) sequencing to investigate the origins of genome size change within the group by identifying signals of WGDs and quantify the abundance of repetitive elements, and (iv) generated a nuclear gene-based phylogenetic framework for the group enabling comparison with the mtDNA-based tree and to act as a backbone for the RAD-based analysis.

## 2. Material and methods

### (a) Phylogeny and genome size analysis

#### (i) Taxonomic sampling and phylogenetic analyses

A total of 221 taxa were included in the analysis consisting of 206 Callichthyidae, including three Callichthyinae (Genera: *Hoplosternum* and *Dianema*), and all known lineages of the Corydoradinae (Genera: *Aspidoras*, *Scleromystax* and *Corydoras*). Six additional outgroup siluriforme taxa (representatives of the Aroidae, Ictaluridae and Claridae), two Characidae, two Gonorynchidae, two Cyprinidae, one Cobitidae, one Catostomidae and one Clupeidae were also included for the fossil dating analysis. We have covered 70% of the described *Corydoras* species, 71% of *Scleromystax*, 100% of *Brochis* and 38% of *Aspidoras*. Voucher information and GenBank accession numbers are provided (electronic supplementary material, table S1).

A 2668 bp mitochondrial dataset (containing partial sequences of 12S rRNA, 16S rRNA, ND4, tRNAHIS, tRNASER and Cytochrome b) was used to construct an ultrametric tree. We used the uncorrelated lognormal relaxed clock method implemented in Beast v. 2.4.7 [21] to estimate divergence times. We calibrated our phylogeny using 6 fossil calibration points (electronic supplementary material, table S2). Beast runs were conducted under a birth–death prior, partitioned using site model averaging implemented in the Beast plugin bModelTest [22]. Four independent MCMC chains were run for 500 million generations, sampling every 50 000 generations starting from a random starting tree. The independent runs were then combined using LogCombiner v. 2.4.7 (http://beast.bio.ed.ac.uk/logcombiner) and inspected for adequate mixing of parameters (ESS > 200) using Tracer v. 1. 6.0 (http://beast.bio.ed.ac.uk/tracer). We then built maximum clade credibility trees with mean node heights using TreeAnnotator v. 2.4.7. Trees were visualized using FigTree v. 1.4.0 (http://beast.bio.ed.ac.uk/figtree) with node ages and 95% highest posterior density (HPD) estimates for divergence times (electronic supplementary material, figure S1). Subsequently, the dated phylogeny was trimmed to include only tips that had genome size estimates from the current study or previously published data for the group obtained from http://www.genomesize.com/.

#### (ii) Genome size estimation and analysis

C-values were estimated from erythrocyte nuclei for 65 species (electronic supplementary material, table S1). Air-dried blood smears were prepared and stained according to a widely used vertebrate protocol [23] using standards from: *Gallus domesticus, Betta splendens, Poecilia reticulata, Chromobotia macracanthus, Danio rerio* and *Polypterus birchir*. Measurements of nuclear area and IOD (integrated optical density) were made using a PriorLux microscope at 100× magnification mounted with a Retiga 2000R CCD camera, and analysed with Image-Pro plus 7 software. C-values were estimated for approximately 100 non-overlapping nuclei from up to five different fields per slide. Genome size estimates for all other available species of Callichthyidae were taken from the Genome Size Database (http://www.genomesize.com/) (electronic supplementary material, table S1). Genome sizes were then mapped onto a trimmed mtDNA phylogeny (only tips with genome sizes retained in the tree) using the Contmap function of the R package *phytools* [24]. The R package *l1ou* [25] was used to investigate whether there was evidence for shifts in genome size using the mtDNA tree. L1ou uses the LASSO (least absolute shrinkage and selector operator) to identify trait shifts and the method does not require predetermination of the number or placement of shifts. Ornstein-Uhlenbeck methods have been shown to be powerful even when the number of taxa are low, provided effect sizes are large [26]. Genome size analyses were conducted using the Bayesian information criterion (BIC) as a model selection criterion, which the authors suggest offers a good compromise between minimizing false positives and maximizing recall rate [25]. To assign a confidence level to each of the detected shifts, non-parametric bootstrapping was used which calculates phylogenetically uncorrelated standardized residuals for each node. These residuals were then sampled with replacement and mapped back onto the tree to create bootstrap replicates.

### (b) Causes of genome size changes

#### (i) RAD library construction and bioinformatic pipeline

For mtDNA lineages 1–8, one species per lineage was selected for RAD sequencing, with two for lineage 9 where genome sizes are highest. *Megalechis* sp. (*Callichthyidae*) was used as the outgroup. Two individuals were used for all species, except for the outgroup where only one sample was available. DNA was extracted using the Qiagen DNA Blood & Tissue Extraction Kit. All samples were treated with RNase and were selected for high quality and high molecular weight by spectrometry and agarose gel electrophoresis, respectively.

The RAD library preparation protocol followed the methodology comprehensively detailed in Etter *et al*. [27], with minor modifications described in Houston *et al*. [28]. Detailed methodology can be found in the electronic supplementary material, Methods.

Raw sequences were cleaned using Trimmomatic [29] using the following settings: LEADING:10 SLIDINGWINDOW:4:20 MINLEN:40. Cleaned data were then imported into CLC Genomics Workbench version 7.0 (CLC, Aarhus, Denmark) and de-multiplexed by barcode identity (Genbank SRA SAMN08384409 - SAMN0838442) and assembled into contigs using Velvet version 1.2.10 [30] (see electronic supplementary material, methods for detailed methods). Sequencing statistics are detailed in electronic supplementary material, table S4.

#### (ii) Detection of whole genome duplication events

To establish whether changes in genome size expansion could be indicative of polyploidy, we searched for signals of WGD in the RAD sequencing data using two-sequence-based methods: haplotype diversity per contig and bi-allelic SNP frequency distribution.

For both of these sequence-based methods, only putative coding regions were used to avoid noise. Contigs were first masked using Repeatmasker version 4.0 [31], before Blastx [32] was used to identify coding regions using default parameters and the nr (non-redundant protein sequences) database. Raw reads for all species were mapped back to these masked contigs using the BWA-mem algorithm (Burrow–Wheeler–Alignment) [33]. A contig was considered correctly assembled if both forward and reverse read of a read-pair map back to the same contig. These ‘verified’ contigs were then used for all further downstream analyses.

WGD events should cause a detectable increase in haplotype diversity at individual contigs and additionally cause a shift in SNP read ratios (a SNP would be covered by a different proportion of reads in a diploid versus a tetraploid). In wheat, 50–60% of homeologues have been shown to be collapsed into single chimeric contigs [34]. In an allopolyploid or a rediploidizing autopolyploid (where duplicated chromosome sets are reverting from tetrasomic to disomic inheritance), these ohnologous regions might be so divergent that they assemble into separate contigs. These contigs would then appear diploid-like using both methods. This should, however, lead to a detectable overall increase in coding contigs which should be identifiable as ohnologues using Blast, for example. In the absence of a reference genome, it is impossible to distinguish between allopolyploidy and autopolyploidy with confidence.

We quantified the number of different haplotypes for each putatively coding contig using Hapler v. 1.60 which performs haplotype calling in low-diversity, low-coverage short-read sequence data [35]. As haplotype assembly can be complicated by reads mapping to consecutive stretches of DNA that do not fully overlap, the data were also filtered to include only haplotypes with a minimum of 20 reads and exclude all alignments that stretch beyond 200 bases. Haplotype numbers per contig in each sample were extracted from the Hapler output and summarized (electronic supplementary material, table S6). Contigs were then grouped according to haplotype number and frequencies were calculated.

As a second method to identify WGDs read count ratios for bi-allelic SNPs were calculated as outlined by Yoshida *et al*. [36]. This method is based on the expectation that mean read ratios for bi-allelic SNPs should differ between samples with different ploidy. For example, in a diploid organism, ratios of the reference reads/non-reference reads are expected to be 1 : 1 (i.e. half of the raw reads should be reference SNP and half should be non-reference). In a triploid, read ratios are expected to be 1 : 2 and in a tetraploid either 1 : 3 or 1 : 1 depending on the progenitor genomes. Thus, in frequency histograms of bi-allelic SNP read ratios we expect a single peak at 0.5 in diploids and peaks at 0.25, 0.5 and 0.75 in tetraploids. Freebayes [37] was used to call polymorphisms with a minimum SNP occurrence of ten reads on each sample replicate. For each sample, resulting datasets were further filtered to contain only bi-allelic SNPs, a maximum total depth of 300 and a minimum reference-allele read count of 5 per individual replicate. SNPs shared between both replicate-libraries were considered real and read counts for the reference and alternate SNPs of both replicates were combined. Histograms of SNP read ratios were plotted per individual and the R package *mixtools* [38] was then used to identify the underlying approximately Gaussian distributions in each histogram. A *k* = 3 was used for each with a starting mu of 0.25, 0.5 and 0.75 and a sigma of 0.05 for each of the three distributions. The relative peak heights of the fitted distributions (lambda) were then used to calculate a 0.25 and 0.75 to 0.5 peak read ratio (height of 0.5 peak/average height of 0.25 + 0.75 peak). These were averaged across the two individuals per species. The read ratio histograms for each individual and associated Gaussian curves are shown in electronic supplementary material, figure S4.

#### (iii) Transposable element identification and quantification

To quantify the relative abundance of TEs in each species, we first de-replicated all raw reads using Usearch [39] with the ‘derep_fulllength’ option before identifying and quantifying repeats and TEs for each species using Repeatmasker with default settings and specifying ‘teleost species’ as the target group [31]. In addition to identifying the main super-families of TEs, we further analysed the Repeatmasker output to quantify Repeat-Classes and Repeat-Families using MS Excel.

#### (iv) Phylogenetic analysis

As the previous phylogeny for the group was generated using mtDNA markers [20], the RAD markers were used to construct a nuclear-based phylogeny using pyRAD [40]. pyRAD filters out potentially paralogous sequences by identifying contigs with more than a set number of heterozygous sites (default = 5) and with a heterozygous site shared between a set number of samples (default = 3). pyRAD also discards clusters with more than two haplotypes. jModeltest [41] was used to determine the most appropriate model of nucleotide substitution (GTR + I + G) before ML and BI trees were constructed using RAXML 8.2.1.0 [42] and MrBayes 3.6 [43]. Two separate MCMC runs were conducted in MrBayes and run for 5 million generations with random starting trees sampling every 500 generations. For RAXML, 1000 rapid bootstrap searches were performed using the Rapid Bootstrapping algorithm. To assess tree concordance across the RAD loci, we used BUCKy [44]. First, we split the concatenated RAD alignment into 1000 bp alignments (approx. 7 RAD loci per alignment). Subsequently, we built individual Bayesian trees using MrBayes 3.6 (5 million generations, GTR + gamma model, 4 chains, 2 independent runs), and processed the resulting tree files for each 1000 bp alignment independently using the BUCKy mbsum utility using a 20% burnin. Individual alignment input files were then run in BUCKy (10 million iterations, using values for the discordance prior of A = 1 and 25).

#### (v) Identification of shifts in trait values

The R package l1ou [25] was again used to investigate whether there was evidence for shifts in magnitude of multi-copy haplotypes, SNP frequency ratio and TE abundance using the tree derived from the RAD data and also, as a comparison, a trimmed mtDNA tree. The RAD tree was made ultrametric by applying non-parametric rate smoothing using the chronos function of the R package ape [45] and scaling the tree to 1. BIC was used as the model selection criterion for all analyses.

## 3. Results

### (a) Chronogram and genome size analysis

**Figure 1.**
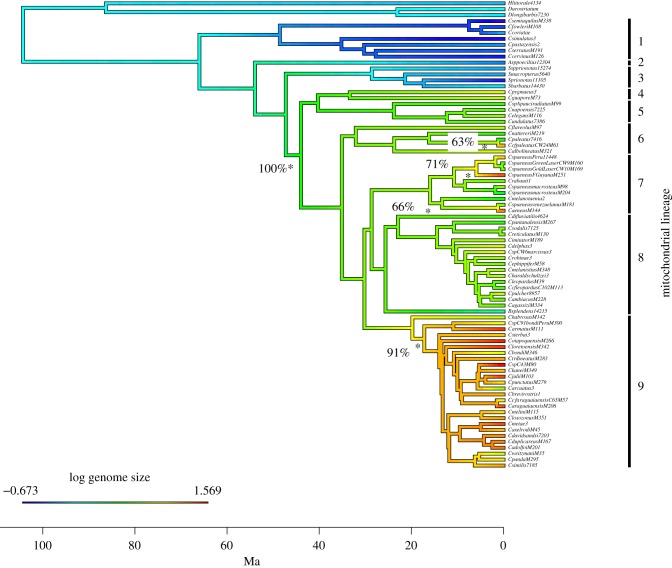
Fossil calibrated chronogram with ln genome size for each species mapped on to the mtDNA tree in colour. Time axis shown in million years ago (Ma). Statistical shifts in genome size are marked with asterisks with associated bootstrap support.

To provide a framework for the investigation of genome size evolution in the Callichthyidae, we generated a time-calibrated mtDNA-based phylogeny using Beast (electronic supplementary material, figure S1). The phylogeny identified nine monophyletic lineages, with most well supported by posterior probabilities greater than 0.9. The most recent common ancestor (mrca) of the Callichthyidae was estimated to be 104 Ma (95% HPD 72.56–132.82 Ma) with the mrca of the Corydoradinae at 66 Ma (95% HPD 55.46–99.5 Ma). We estimate the mrca of the Siluriformes to be 139 Ma (95% HPD 98.07–173.36 Ma). The ages estimated in the current study are somewhat older than dates published previously for the Callichthyidae: Mariguela *et al.* [46] used a single fossil calibration for the stem of the Callichthyidae at 58 Ma. However, our dates for other non-Callichthyidae nodes are concordant with other studies e.g. the origin of the Siluriformes, which has been previously estimated to be between 100 and 145 Ma [47,48]. The phylogenetic tree was then trimmed to include only tips where genome size information had been generated (figure 1).

#### (i) Genome size estimation

Haploid genome sizes (C-values) ranged between 0.51 and 4.8 pg (figure 1, electronic supplementary material, table S1). Lineages 1, 2 and 3 exhibited C-values ranging between 0.51 and 0.94 pg (mean 0.71 ± 0.13), followed by lineages (4–8) which showed higher average genome size and higher variation among taxa within a lineage. The largest average C-values were identified in lineage 9 at 4.8 pg, which is the largest genome size of any recorded teleost fish. While the averages were highest in lineage 9, lineages 6 and 7 also had individual taxa with high genome sizes (figure 1). Five shifts in C-values were identified (figure 1) using the R package *l1ou* which uses an Ornstein-Uhlenbeck model-based process to identify shifts in trait magnitude, the first occurring at the stem of lineages 4–9 (100% bootstrap support) dated at a maximum of 44–47 Ma (95% HPD 36.2–68 Ma). A second major shift was detected close to the base of lineage 9 (87% bootstrap support) with an age of approximately 19 Ma (95% HPD 15.3–30.14 Ma). Three additional single branch shifts were identified: one within lineage 6 and two within lineage 7 (65%, 77% and 66% bootstrap support, respectively) (figure 1).

### (b) Causes of genome size changes

#### (i) RAD sequencing

The first sequencing run yielded roughly 104 million paired reads (GC content 47%). After quality filtering and trimming, 93.52% of the original sequences remained. The second sequencing run resulted in roughly 117 million paired sequences (GC content 46%), with 81.99% of paired sequences surviving filtering steps. The number of contigs assembled for each species ranged between 13 166 (*C. aeneus*) and 58 604 (*C. imitator*), with N50 ranging from 270 (*C. nattereri*) to 447 bp (*C. imitator*) (electronic supplementary material, table S4).

#### (ii) RAD sequence-based phylogeny

**Figure 2.**
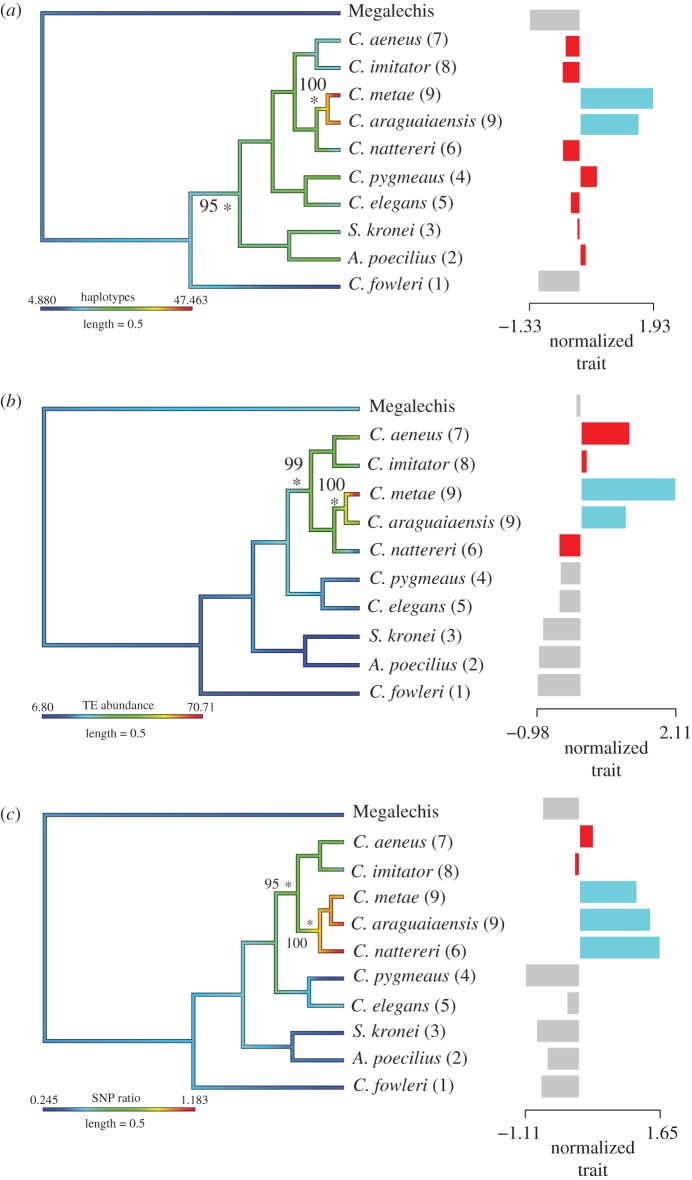
Phylogenetic trees based on RAD sequence data with lineage in parenthesesm: (*a*) haplotypes per contig, (*b*) TE abundance and (*c*) SNP read ratio per contig mapped on in colour. Stars indicate nodes where shifts in trait values occur with bootstrap support. The right-hand panel shows the positive and negative shifts in trait size identified by l1ou. Different colours in right-hand panels indicate shifts in magnitude of trait.

The conservative concatenated dataset generated by pyRAD consisted of 44 521 bases of sequence which contained 7879 variable sites, 5591 of which were parsimony informative, with 5.9% missing data across all taxa. Both the Bayesian and maximum likelihood methods identified a single tree topology with high support for all branches (electronic supplementary material, figure S2a). The topology of this nDNA-based tree was almost identical to that of the previously published mtDNA-based tree [20] with one exception: lineage 6 shared a common ancestor with lineage 9, whereas in the mtDNA tree it was basal to lineages 7, 8 and 9 (figures 1 and 2). The discordance analysis showed a concordance metric of 1 for the clade with lineage 6 and lineage 9 (electronic supplementary material, figure S2b), suggesting the phylogenetic signal supporting a single clade including lineage 6 and 9 was present across the sampled loci.

#### (iii) Detection of whole genome duplication events using RAD data

**Figure 3.**
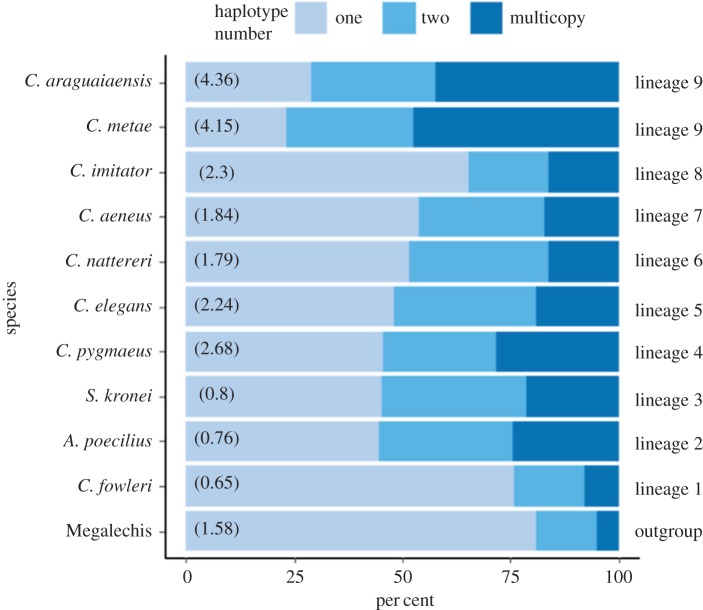
Average haplotype abundance per contig for the Corydoradinae lineages. Genome size displayed in parentheses. (Online version in colour.)

There were marked differences in the number of haplotypes identified per assembled contig across species. For two of the assumed diploid lineages *Megalechis* (outgroup) and *C. fowleri* (lineage 1), more than 95% of contigs had one or two haplotypes, with very few multi-copy contigs (figures 2*a* and 3; electronic supplementary material, table S6). There was a reduction in the proportion of contigs with one haplotype (from more than 75% down to around 50% depending on lineage) and a parallel increase in contigs with two or multiple haplotypes in lineage 2 to lineage 8. In the two lineage 9 species a further jump in multicopy haplotypes was identified, with almost half of all contigs exhibiting two or multiple haplotypes (figure 3). Two shifts in magnitude in haplotype number per contig were detected on the RAD and mtDNA tree analysis using *l1ou*, the first at the stem of lineage 2–9 (RAD = 95% bootstrap support, mtDNA = 95% support) at between 54 and 66 Ma (95% HPD 42.56–99.5 Ma) and the second at the stem of lineage 9 (RAD and mtDNA = 100% bootstrap support) at 20–30 Ma (95% HPD 15.3–45 Ma) (figure 2*a*; electronic supplementary material, figure S4). An additional increase in haplotype number was detected in the mtDNA tree in lineage 4 (95% bootstrap support) (electronic supplementary material, figure S4). Overall, there was no detectable increase in putatively coding contigs with higher genome size (electronic supplementary material, table S6), a pattern that would have been expected if putative ohnologues were assembled into separate contigs. The detected increase in haplotypes per contig in the absence of an increase in contig number suggests that duplicated genes (homeologues) were predominantly assembled into single contigs.

The SNP frequency distribution analysis revealed that both *Megalechis* (outgroup) and *C. fowleri* (lineage 1) displayed a clear peak around 0.5, i.e. the majority of bi-allelic SNPs have roughly an even read number as expected in a diploid species (electronic supplementary material, figure S3). Species in lineages 2–8 all display a large peak at 0.5 with slight differences in distribution. Most species also display small peaks at 0.25 and 0.75 frequencies, which may be a result of tandemly duplicated genes, and multi-gene families which may make up more than 30% of protein coding genes even in diploids [49,50]. We were unable to filter against these putative paralogues without also filtering out ohnologues in the absence of a reference genome. Visual investigation of read ratios within the dataset revealed that species in lineages 1–4 (and outgroup) displayed a strong peak at 0.5 with relatively small peaks at 0.25 and 0.75 with ratios between 0.25 and 0.4. A second group displayed ratios between 0.53 and 0.72, while a final group had ratios of 1.02–1.18, in which the 0.25 and 0.75 peaks were the same size or larger than the 0.5 peaks (figure 2*c*). In a functional tetraploid, SNP read ratios are expected to display peaks at a 0.5 read ratio and at 0.75/0.25. Thus *C*. *araguaiaensis* and *C. metae* (Lineage 9), *C. nattereri* (Lineage 6) displayed SNP frequency distributions that were consistent with tetraploidy and lineages 5, 7 and 8 display some evidence of an older duplication event. Two shifts in SNP ratio were detected using *l1ou*, in the RAD tree analysis an increase at the stem of lineage 6,7,8,9 at between 35 and 44 Ma (95% HPD 30.29–64.51 Ma) and an increase at the stem the clade containing lineages 6 and 9 at between 20 and 30 Ma (95% HPD 15.3–45 Ma) (assuming lineage 6 is part of lineage 9). In the mtDNA dataset, two shifts were also detected, one at the stem of lineages 6–9 (aged at between 30 and 35 Ma (95% HPD 24.67–54.33 Ma)) and a decrease at the stem of lineages 7 and 8 with an age of 29–30 Ma (95% HPD 23.18–45 Ma).

#### (iv) Transposable element identification and quantification

Repeatmasker revealed large differences in repetitive element abundance among species. Lineages with larger genome sizes had a higher abundance of repetitive elements (figure 2*b*). TE abundance was stable across lineages 1–3 with approximately 10% of sequences containing TEs. There was an increase in TE abundance across lineages 4–6 which have more than 20% TE content. A second increase in TE abundance occurred at the stem of lineage 7 (*C. aeneus*) with more than half the data comprising repetitive elements—a five times increase when compared with lineage 1. The highest abundance of TE elements occurred in lineage 9 where up to 70% of reads were TEs (figure 2*b*). Shifts in total TE abundance were identified using *l1ou* [25]. Two shifts were identified in the RAD tree dataset, the oldest with an age of 30–44 Ma (95% HPD 30.29–64.51 Ma), and the youngest in the stem of lineages 6 and 9 at between 20 and 30 Ma (95% HPD 15.3–45 Ma). Two shifts were also identified in the mtDNA dataset, one at the stem of lineage 9 with an age of at 20–30 Ma (95% HPD 15.3–45 Ma) and one at the stem of lineages 7 and 8 with an age of 29–30 Ma (95% HPD 23.18–45 Ma). TC1-IS630-Pogo elements appear to have driven the main TE expansion in the Corydoradinae (electronic supplementary material, table S3) with the abundance increasing from less than 1% of the sequences in lineage 1 (*C. fowleri*) to over 70% of the sequences in lineage 9 (*C. metae*) (electronic supplementary material, table S3).

## 4. Discussion

Here, we elucidate for the first time the evolutionary history of genome size change within the Neotropical Corydoradinae catfishes. Two major evolutionary increases in genome size were identified, one at the stem of lineage 4 and a second at the stem (and/or within) of lineage 9 (figure 1). Independent branch-specific genome size shifts were also identified in lineages 6 and 7. RAD sequencing revealed that there have been at least two positive shifts in haplotype diversity per contig and SNP read ratio across the tree which are indicative of WGDs (figure 2). The timing of the oldest WGD event as indicated by RAD analyses based on haplotype diversity is 54–66 Ma (95% HPD 42.56–99.5 Ma) (group including lineages 2–9). SNP read ratio data do not find a shift at the base of lineage 2 but detect a signal congruent with polyploidy at the stem of lineages 6,7,8,9 (aged between 35 and 44 Ma (95% HPD 30.29–64.51 Ma), a pattern that could be explained by post-WGD genome evolution and rediploidization. Both methods agreed on a more recent event associated with lineage 9 (which includes lineage 6 using nuclear data) suggesting that these species may be functionally polyploid with a maximum age of between 20 and 30mya (95% HPD 15.3–45 Ma). TE abundance increased markedly in tandem with genome size increase, with a single family of TEs (TC1-IS630-Pogo) showing two increases across Corydoradinae, one associated with lineage 9, the other at the stem of lineages 7–9.

### (a) Genome downsizing

Following WGD events, genomes typically undergo extensive ‘pruning’ and return to an almost diploid-like state, with only traces of the ancestral duplication event remaining in the genome—a process commonly referred to as rediploidization [51]. One of the key steps in diploidization is the return from multivalent formation to bivalent formation of chromosomes during meiosis—particularly in autopolyploids [51]. This process may be aided through large-scale rearrangements that frequently occur post-WGD [52] which may impair homeologous pairing during meiosis. Allopolyploids may exhibit disomic inheritance rapidly after formation if genetic differences between progenitor species are sufficient to prevent homolous pairing. In allopolyploids, genome downsizing appears to occur within the first few generations after formation [53]. It has recently been shown that genomic rearrangements have played a major role in the rediploidization process of the Atlantic salmon (*Salmo salar*) [6]. This rediploidization process may explain the different patterns identified using the two RAD-based methods, where the haplotype analysis shows a shift in lineage 2 but the SNP read ratio as well as the genome size do not. After rediploidization, when the genome returns to a functionally diploid state following re-establishment of disomic inheritance, we would expect SNP read ratios to be more similar to diploid samples. Concomittantly, as homeologues diverge and are resolved into disomically inherited pairs, contig assembly may still assemble homeologues into chimeric contigs resulting in an increased haplotype count per contig. Our results suggest that lineages 2 and 3 may be paleopolyploids that have rediploidized following a WGD event. The fossil-calibrated phylogeny estimates the age of the oldest WGD at between 54 and 66 Ma (95% HPD 42.56–99.5 Ma) which is younger than the salmonid WGD event estimated to have occurred between 88 and 103 Ma [54]. The salmon lineage is in an advanced stage of the rediploidization process [55], though it has been suggested that this process may have been retarded by the formation of meta-centric chromosomes [56]. It is therefore plausible that *Corydoras* could re-diploidize either partially or fully in this time frame.

By contrast, the additional WGD event or events identified in lineage 9 are much more recent—approximately 19 Ma (95% HPD 15.3–30.14 Ma). With our limited RAD sampling, it is not possible to determine whether the entire lineage has undergone an additional WGD event, or whether this event is restricted to those species with the largest genome sizes which were sampled here (figure 1). SNP read ratios generated from the RAD data for the two lineage 9 species indicate that these may still be functional polyploids.

### (b) Transposable element expansion

TEs have been shown to have had a major impact on genome size across the vertebrates, with genome size correlated with TE content [57]. Teleost fishes have the most diverse TE compliments and also appear to have quite varied TE abundance across species [57]. In this study, the RAD sequencing data identified two increases in DNA transposon abundance across the Corydoradinae, the oldest with an age of 30–44 Ma (95% HPD 30.29–64.51 Ma), and the youngest at the stem of lineages 6 and 9 at between 20 and 30 Ma (95% HPD 15.3–45 Ma). Two shifts were also identified in the mtDNA dataset, one at the stem of lineage 9 with an age of 20–30 Ma (95% HPD 15.3–45 Ma) and one at the stem of lineages 7 and 8 with an age of 29–30 Ma (95% HPD 23.18–45 Ma). The driver of the overall increase was a single DNA transposon family, TC1-IS630-Pogo, which are also the most abundant repeat types in the channel catfish genome (*Ictalurus punctatus*) making up roughly 4–5% of the genome. TC1 elements are particularly common in fish and amphibians [57] but are also found in fungi, plants and ciliates. TC1 elements are typically evenly spread across the genome, whereas other retroelement families may be clustered in specific areas of chromosomes or genes [58]. RAD sequencing (the cut sites of which are spread across the genome) may be biased towards identifying TC1-like elements, and may result in an underestimate of clustered TE-families. While the absolute abundance of TE elements is not quantifiable using RAD data, the relative changes in abundance across the phylogeny are quantifiable and clearly play an important role in genome size increase in lineages 7 and 9.

### (c) Simultaneous whole genome duplication and transposable element expansion?

WGD events and subsequent TE proliferation have previously been linked in rice (*Oryza species*), maize (*Zea mays*) [8] and the evolution of the hugely diverse angiosperms [59]. TEs are likely to be mostly deleterious as a result of insertions interrupting gene activity or regulation [60] and TEs are usually epigenetically silenced for these reasons. However, polyploidy and hybridization may interrupt the suppression mechanisms, allowing TEs to proliferate [59]. In this study, an increase in TE elements does not appear to have coincided with the oldest WGD (stem of lineage 2 or 4), but does appear to be associated with the more recent WGDs in lineage 7 and 9. TE activity may have deleterious consequences for the organism, but TEs may also create genetic variation and this has been implicated in many cases of adaptive evolution, such as adaptation to novel environments, stressors or environmental change [61]. For example, van't Hof *et al*. [16] have shown that the industrial melanism mutation in the British peppered moth was caused by a TE insertion. Expansions of repetitive elements have also been identified in the Salmonidae which underwent a WGD 88–103 Ma. In salmonids, the expansion of the TC1-Mariner family occurred after the WGD, and has been linked with speciation in the group [62]. Moreover, TEs have been suggested to play an important role in the diploidization process as TEs accumulate differentially on duplicated chromosomes in autopolyploids [63]. In the Atlantic salmon, genomic rearrangements which aided the rediploidization process were likely driven by bursts of repeat expansions [6]. In this study, we did not detect a burst of TEs in lineage 2 or 3 and thus found no evidence to suggest TE expansions were involved in the rediploidization of lineages 2 and 3. However, it should be noted that RAD sequencing could miss such a proliferation if changes in the restriction enzyme cut sites occur. TE expansions may also lead to Dobzhansky–Muller incompatibilities between different isolated populations which may increase the rate of attainment of reproductive isolation and thus speciation [64].

While it is acknowledged that genome size does not directly correlate with organismal complexity [1], WGD and TE expansion may have profound consequences for the subsequent evolution of a lineage. Here, we show that genome size in the Corydoradinae is driven by both WGD events and TE expansions, and we provide strong evidence that some Corydoradinae species are polyploids. Our findings open up an exciting set of questions for evolution and adaptation in relation to both WGD and TEs, and we propose that Corydoradinae make an excellent study system with which to disentangle effects of both WGD and TE expansion on adaptive evolution.

## Supplementary Material

Supplementary methods

## Supplementary Material

Supplementary tables and figures

## Data accessibility

Demultiplexed rad sequences: GenBank SRA SAMN08384409-SAMN0838442. Beast xml file for Corydoradinae chronogram: http://dx.doi.org/10.5061/dryad.8108d [65].
