# Supplementary material for: Whole genome duplication and transposable element proliferation drive genome expansion in Corydoradinae catfishes
Source: Proc Biol Sci. 2018 Feb 14;285(1872):20172732. doi: 10.1098/rspb.2017.2732 (PMC5829208; doi:10.1098/rspb.2017.2732)
Supplement: Supplementary methods [file rspb20172732supp1.docx]

**Detailed RAD Library methods**

Replicate digests (n=2) were performed per DNA sample, with different combinations of barcoded adapters then ligated to each digested sample. Briefly, 600 ng DNA was individually digested with 4 U SbfI high fidelity restriction enzyme (New England Biolabs; NEB) in 1× Reaction Buffer 4 (NEB) at 37°C for 45 minutes. The reactions (30 µL final volumes) were then heat inactivated at 65°C for 20 minutes. Individual specific P1 adapters, each with a unique 5 or 7 base barcode (Supplementary Table 5; variable size to increase sequence complexity through the RE site), were ligated to the SbfI digested DNA, at 22°C for 60 minutes, by adding 1.5 µL 100 nM P1 adapter, 0.36 µL 100 mM rATP (Promega), 0.6µL 10× Reaction Buffer 2 (NEB), 0.3 µL T4 ligase (NEB, 2 M U/mL) and reaction volumes made up to 36 µL volume with nuclease free water. Following ligation, the samples were heat inactivated at 65°C for 20 minutes, cooled to room temperature, then combined into species-specific library pools (Supplementary Table 5; generally four samples per species). Shearing (Covaris S2 sonication) and initial size selection (c. 200–500 bp) by agarose gel separation of both library pools was followed by gel purification, end repair, dA overhang addition, P2 paired-end adapter ligation and library amplification, exactly as in the original RAD protocol [[3](#_ENREF_3)]. Either a single P2 adapter or equimolar combinations of two P2 adapters with 5 and 6 base barcodes (1µL of 10µM P2 adapter mix per library) was used for each species pool. The unique combination of P1 and P2 barcodes allowed identification of each sample post sequencing. A total of 200 µL of each amplified library was produced (16× 12.5 µL reactions; × 14 PCR cycles) was prepared and size selected (c. 320–650 bp) by gel electrophoresis. Following a final gel elution step into 20 µL EB buffer (MinElute Gel Purification Kit, Qiagen), the libraries were quantified by fluorimetry. Sequencing of species specific libraries was carried out at the Earlham Institute (formerly TGAC), Norwich; Illumina HiSeq2000 platform, 150 base paired-end reads. Two lanes of sequencing were performed with different combinations of species in each run (Supplementary Table 5). In order to achieve approximately similar read coverage per locus, the molar ratios of species-specific libraries in each run were adjusted to reflect their previously estimated genome size.

*Contig assembly*

Paired-end reads were assembled separately for each species using Velvet version 1.2.10 [[4](#_ENREF_4)]. The wrapper script Velvetoptimiser version 2.2.5 [[5](#_ENREF_5)] was used to optimize the three parameters: k (word length), expected coverage and coverage cutoff. The optimisation function used was the default N50. In cases where assembly was difficult, the optimisation function was changed to number of contigs. Because RAD data coverage is expected to be highly non-uniform and k-mer coverage in Velvet was highly variable after first optimization attempts, we normalized the coverage across contigs using the digital normalization script bbNorm which is part of the bbMap package (http://sourceforge.net/projects/bbmap/). Reads of all samples (including replicates) were combined per species for the assembly. As reverse reads vary more in coverage (due to the random shearing step) as well as to further facilitate the assembly of repetitive regions, reverse reads were assembled in a separate run using a longer kmer length. These contigs were then passed back into the complete assembly as long reference reads. A smaller k was used for this step to aid connecting the forward and reverse reads. The mean read depth was calculated using the GATK Depth of Coverage Tool [[6](#_ENREF_6)]. Sequencing statistics are detailed in Supplementary Table 3.

**References**

[1] Houston, R.D., Davey, J.W., Bishop, S.C., Lowe, N.R., Mota-Velasco, J.C., Hamilton, A., Guy, D.R., Tinch, A.E., Thomson, M.L., Blaxter, M.L., et al. 2012 Characterisation of QTL-linked and genome-wide restriction site-associated DNA (RAD) markers in farmed Atlantic salmon. *Bmc Genomics* **13**, 244. (doi:10.1186/1471-2164-13-244).

[2] Baxter, S.W., Davey, J.W., Johnston, J.S., Shelton, A.M., Heckel, D.G., Jiggins, C.D. & Blaxter, M.L. 2011 Linkage mapping and comparative genomics using next-generation RAD sequencing of a non-model organism. *Plos One* **6**, e19315. (doi:10.1371/journal.pone.0019315).

[3] Etter, P.D., Bassham, S., Hohenlohe, P.A., Johnson, E. & Cresko, W.A. 2011 SNP Discovery and Genotyping for Evolutionary Genetics Using RAD Sequencing (eds. M.V. Orgogozo & M.V. Rockman).

[4] Zerbino, D.R. & Birney, E. 2008 Velvet: Algorithms for de novo short read assembly using de Bruijn graphs. *Genome Res* **18**, 821-829. (doi:10.1101/gr.074492.107).

[5] Gladman, S. & Seeman, T. 2012. VelvetOptimiser. . (

[6] McKenna, A., Hanna, M., Banks, E., Sivachenko, A., Cibulskis, K., Kernytsky, A., Garimella, K., Altshuler, D., Gabriel, S., Daly, M., et al. 2010 The Genome Analysis Toolkit: A MapReduce framework for analyzing next-generation DNA sequencing data. *Genome Res* **20**, 1297-1303. (doi:10.1101/gr.107524.110).
