# Supplementary material for: Whole genome duplication and transposable element proliferation drive genome expansion in Corydoradinae catfishes
Source: Proc Biol Sci. 2018 Feb 14;285(1872):20172732. doi: 10.1098/rspb.2017.2732 (PMC5829208; doi:10.1098/rspb.2017.2732)
Supplement: Supplementary tables and figures [file rspb20172732supp2.docx]

** Supplementary Figure 1. Fossil calibrated phylogeny of Corydoradinae. Node ages and 95% HPD shown as blue bars. Fossil calibration points are numbered with siluriform fossils in red.**

Supplementary Figures and Tables


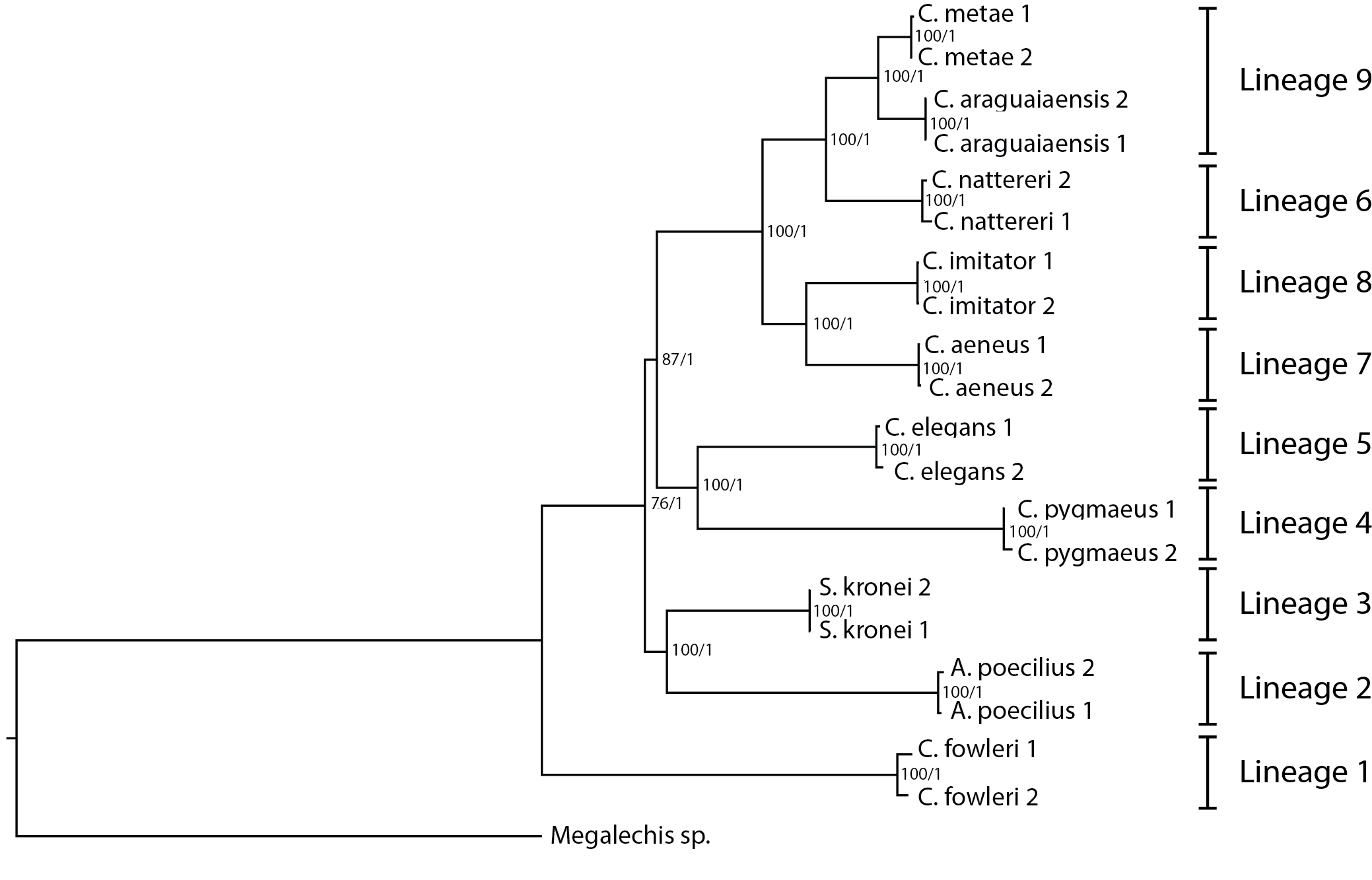


**Supplementary Figure 2a**. Phylogenetic tree constructed using ML (RAXML) and Bayesian (MrBayes)

Methods from the concatenated RAD data set. Bootstrap support and posterior probabilities are indicated for each node.


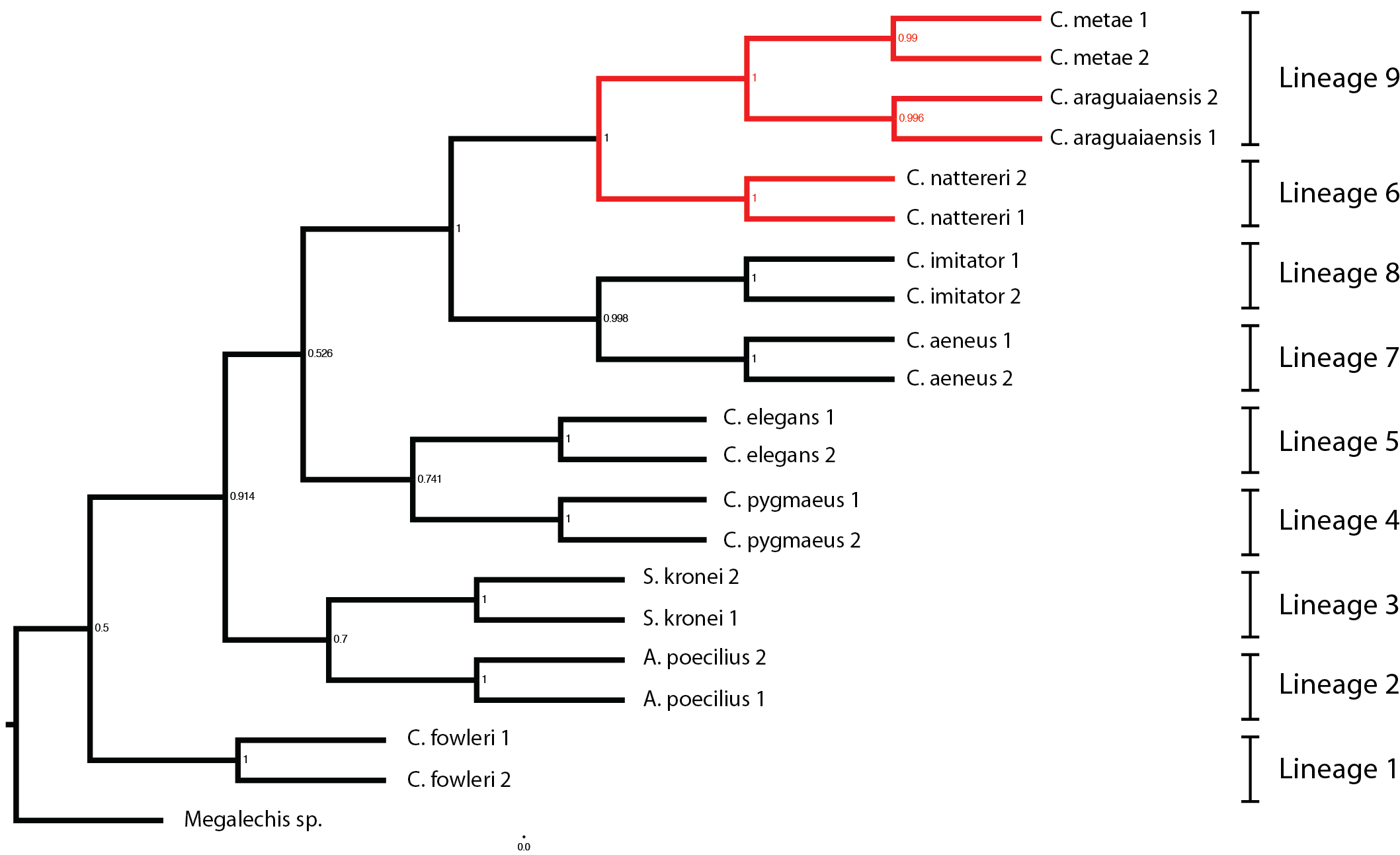


**Supplementary Figure 2b**. Bucky concordance analysis using RAD data showing Bucky concordance metric across RAD loci. Lineage 6 and 9 are marked in red.


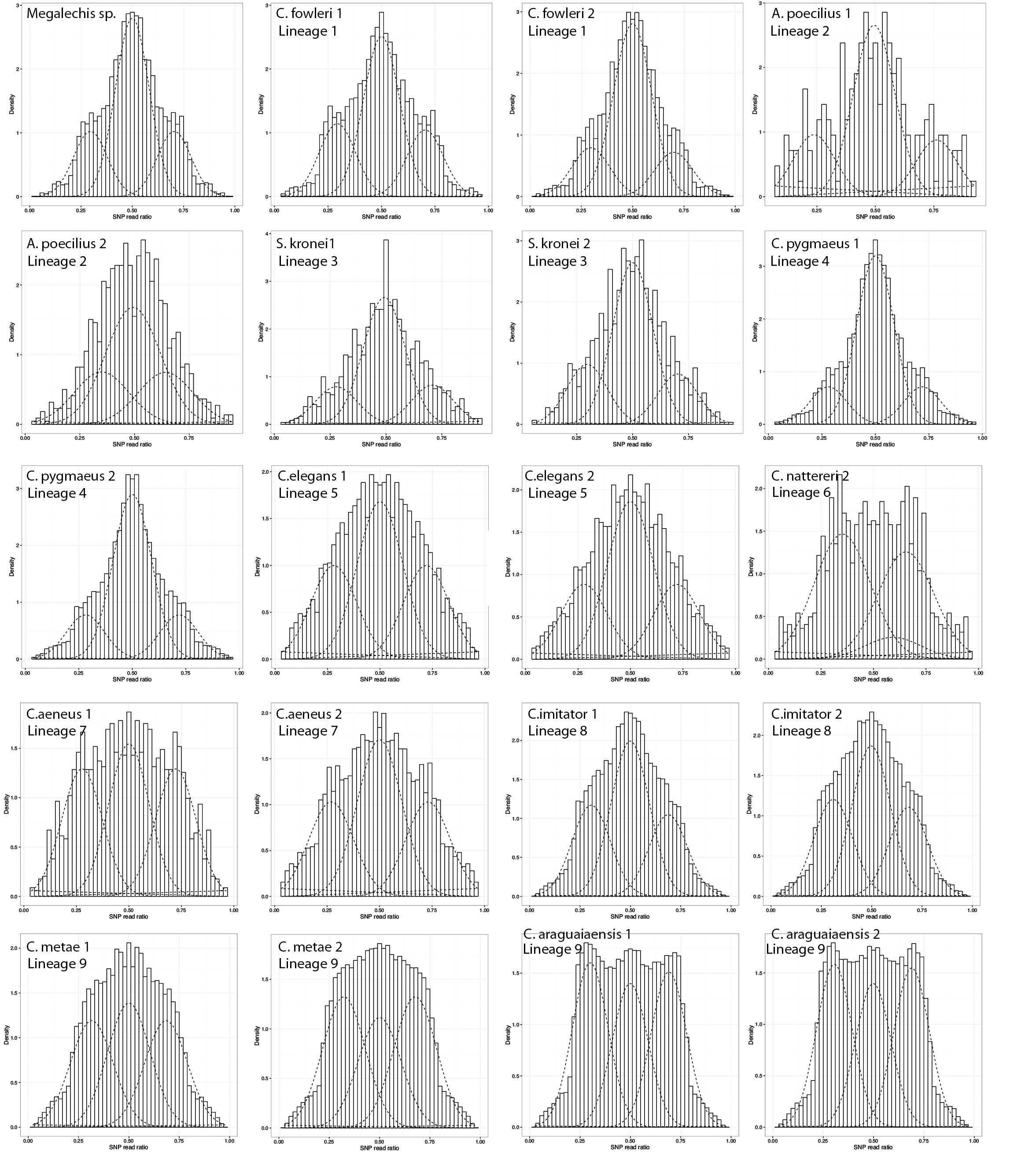


**Supplementary Figure 3.** Histograms of SNP read ratios per individual. The Gaussian mixed model peak heights for K=3 are shown overlaid with dotted lines.

Supplementary Figure 4. Statistical shifts in haplotype number per contig, (i) Transposable element abundance (ii) and SNP read count ratio (iii) using mtDNA based tree. Lineages are shown in parentheses after taxon name.

**Supplementary Table 1**. Accession numbers and Genome size data for Corydoradinae and outgroups used in current study.

| **Subfamily** | **Lineage** | **Genus** | **Species** | **Voucher Code** | **Hap C-value (pg)** | **ACC 12s** | **ACC 16s** | **ACC ND4** | **ACC Cytb** |
| --- | --- | --- | --- | --- | --- | --- | --- | --- | --- |
| Callichthyinae | Lineage 0 | *Dianema* | *D. longibarbus* | LBP 557-7230 |  | GU210442 | GU210867 | GU210020 | GU209684 |
| Callichthyinae | Lineage 0 | *Dianema* | *D. urostriatum* | MT89 | 0.99 | GU210539 | GU210964 | GU210114 | GU209685 |
| Callichthyinae | Lineage 0 | *Hoplosternum* | *H. littorale* | LBP 210-4134 | 0.85 | GU210443 | GU210868 | GU210021 | GU209686 |
| Corydoradinae | Lineage 1 | *Corydoras* | *C. acutus* | MA41 |  | GU210339 | GU210764 | GU209918 | GU209605 |
| Corydoradinae | Lineage 1 | *Corydoras* | *C. amapaensis* | MA154 |  | GU210164 | GU210589 | GU209745 | GU209360 |
| Corydoradinae | Lineage 1 | *Corydoras* | *C. aurofrenatus* | ANSP 182420-1470 |  | GU210327 | GU210752 | GU209907 | GU209332 |
| Corydoradinae | Lineage 1 | *Corydoras* | *C. blochi* | MHNG 2652.007-GY04-237 |  | GU210236 | GU210661 | GU209816 | GU209341 |
| Corydoradinae | Lineage 1 | *Corydoras* | *C. cervinus* | MA125 | 0.6 | GU210146 | GU210571 | GU209727 | GU209358 |
| Corydoradinae | Lineage 1 | *Corydoras* | *C.* cf*. blochi* | MHNG 2707.015-SU07-624 |  | GU210239 | GU210664 | GU209819 | GU209368 |
| Corydoradinae | Lineage 1 | *Corydoras* | *C.* cf*. geoffroy* | MHNG 2683.016-GF06-459 |  | GU210249 | GU210674 | GU209829 | GU209379 |
| Corydoradinae | Lineage 1 | *Corydoras* | *C.* cf*. maculifer* | MA40 |  | GU210338 | GU210763 | GU209917 | GU209386 |
| Corydoradinae | Lineage 1 | *Corydoras* | *C. coriatae* | MT14 | 0.81 | GU210428 | GU210853 | GU210006 | GU209408 |
| Corydoradinae | Lineage 1 | *Corydoras* | *C. ellisae* | MT24 |  | GU210469 | GU210894 | GU210047 | GU209430 |
| Corydoradinae | Lineage 1 | *Corydoras* | *C. fowleri* | MA108 | 0.65 | GU210133 | GU210558 | GU209715 | GU209441 |
| Corydoradinae | Lineage 1 | *Corydoras* | *C. geoffroy* | MHNG 2700.007-GF07-120 |  | GU210226 | GU210651 | GU209806 | GU209443 |
| Corydoradinae | Lineage 1 | *Corydoras* | *C. maculifer* | LBP 7213-32890 |  | GU210210 | GU210635 | GU209790 | GU209481 |
| Corydoradinae | Lineage 1 | *Corydoras* | *C. negro* | MA52 |  | GU210348 | GU210773 | GU209927 | GU209504 |
| Corydoradinae | Lineage 1 | *Corydoras* | *C. orcesi* | MA304 |  | GU210293 | GU210718 | GU209873 | GU209512 |
| Corydoradinae | Lineage 1 | *Corydoras* | *C. oxyrhynchus* | MHNG SU08-1191 |  | GU210284 | GU210709 | GU209864 | GU209516 |
| Corydoradinae | Lineage 1 | *Corydoras* | *C. pastazensis* | MT42 | 0.78 | GU210488 | GU210913 | GU210066 | GU209527 |
| Corydoradinae | Lineage 1 | *Corydoras* | *C. semiaquilus* | ANSP 178613-1459 | 0.51 | GU210321 | GU210746 | GU209901 | GU209553 |
| Corydoradinae | Lineage 1 | *Corydoras* | *C. septentrionalis* | MA114 |  | GU210139 | GU210564 | GU209721 | GU209659 |
| Corydoradinae | Lineage 1 | *Corydoras* | *C. serratus* | MA309 | 0.7 | GU210298 | GU210723 | GU209878 | GU209557 |
| Corydoradinae | Lineage 1 | *Corydoras* | *C. simulatus* | MT64 | 0.52 | GU210512 | GU210937 | - | GU209566 |
| Corydoradinae | Lineage 1 | *Corydoras* | *C. solox* | MHNG 2666.036-GF03-099 |  | GU210252 | GU210677 | GU209832 | GU209573 |
| Corydoradinae | Lineage 1 | *Corydoras* | *C.* sp*.* 'C115' | MA183 |  | GU210186 | GU210611 | GU209767 | GU209604 |
| Corydoradinae | Lineage 1 | *Corydoras* | *C.* sp*.* 'C42' | MA27 |  | GU210263 | GU210688 | GU209843 | GU209625 |
| Corydoradinae | Lineage 1 | *Corydoras* | *C.* sp*.* 'C53' | MA30 |  | GU210288 | GU210713 | GU209868 | GU209630 |
| Corydoradinae | Lineage 1 | *Corydoras* | *C.* sp. 'CW11 long nose *reynoldsi'* | LBP 7712-32724 |  | GU210203 | GU210628 | GU209783 | GU209640 |
| Corydoradinae | Lineage 1 | *Corydoras* | *C.* sp*. amapaensis* | MHNG 2681.018-GF06-071 |  | GU210256 | GU210681 | GU209836 | GU209318 |
| Corydoradinae | Lineage 1 | *Corydoras* | *C.* sp*.* ‘C109’ | LBP 5549-27241 |  | GU210130 | GU210555 | GU209712 | GU209653 |
| Corydoradinae | Lineage 1 | *Corydoras* | *C.* sp*.* ‘C92’ | MA211 |  | GU210213 | GU210638 | GU209793 | GU209304 |
| Corydoradinae | Lineage 1 | *Corydoras* | *C. stenocephalus* | MHNG PE08-910 |  | GU210279 | GU210704 | GU209859 | GU209554 |
| Corydoradinae | Lineage 1 | *Corydoras* | *C. treitlii* | MT76 |  | GU210525 | GU210950 | GU210100 | GU209663 |
| Corydoradinae | Lineage 1 | *Corydoras* | *C. vittatus* | MT84 |  | GU210534 | GU210959 | GU210109 | GU209677 |
| Corydoradinae | Lineage 2 | *Aspidoras* | *A. albater* | MA329 |  | GU210313 | GU210738 | GU209893 | GU209283 |
| Corydoradinae | Lineage 2 | *Aspidoras* | *A. depinnai* | MA307 |  | GU210296 | GU210721 | GU209876 | GU209285 |
| Corydoradinae | Lineage 2 | *Aspidoras* | *A. eurycephalus* | MA176 |  | GU210180 | GU210605 | GU209761 | GU209286 |
| Corydoradinae | Lineage 2 | *Aspidoras* | *A. microgaleus* | MA153 |  | GU210163 | GU210588 | GU209744 | GU209287 |
| Corydoradinae | Lineage 2 | *Aspidoras* | *A. poecilus* | LBP 1272-11098 |  | GU210542 | GU210967 | GU210117 | GU209288 |
| Corydoradinae | Lineage 2 | *Aspidoras* | *A. raimundi* | LBP 5568 |  | GU210131 | GU210556 | GU209713 | GU209290 |
| Corydoradinae | Lineage 2 | *Aspidoras* | *A.* sp. 'C35 Black Phantom' | MA177 |  | GU210181 | GU210606 | GU209762 | GU209291 |
| Corydoradinae | Lineage 2 | *Aspidoras* | *A.* sp*. poecilus* | MA96-27708 | **0.76*** | GU210379 | GU210804 | GU209957 | GU209284 |
| Corydoradinae | Lineage 2 | *Aspidoras* | *A.* sp*. poecilus* | LBP 1437-12304 |  | GU210544 | GU210969 | GU210119 | GU209294 |
| Corydoradinae | Lineage 2 | *Aspidoras* | *A. spilotus* | MA325 |  | GU210309 | GU210734 | GU209889 | GU209292 |
| Corydoradinae | Lineage 2 | *Aspidoras* | *A. taurus* | MA328 |  | GU210312 | GU210737 | GU209892 | GU209296 |
| Corydoradinae | Lineage 3 | *Scleromystax* | *S. barbatus* | LBP 2083-14430 | 0.94 | GU210446 | GU210871 | GU210024 | GU209689 |
| Corydoradinae | Lineage 3 | *Scleromystax* | *S. kronei* | LBP 2658-17418 | 0.94 | GU210448 | GU210873 | GU210026 | GU209691 |
| Corydoradinae | Lineage 3 | *Scleromystax* | *S. lacerdai* | LBP 1966-13705 |  | GU210452 | GU210877 | GU210030 | GU209695 |
| Corydoradinae | Lineage 3 | *Scleromystax* | *S. macropterus* | LBP 461-5642 | **0.75** | GU210455 | GU210880 | GU210033 | GU209698 |
| Corydoradinae | Lineage 3 | *Scleromystax* | *S. prionotus* | LBP 1267-11106 | **0.6** | GU210457 | GU210882 | GU210035 | GU209700 |
| Corydoradinae | Lineage 3 | *Scleromystax* | *S*. sp. 'C113' | LBP 1237-11125 |  | GU210459 | GU210884 | GU210037 | GU209704 |
| Corydoradinae | Lineage 3 | *Scleromystax* | *S*. sp. 'CW42' | MA112 |  | GU210137 | GU210562 | GU209719 | GU209705 |
| Corydoradinae | Lineage 3 | *Scleromystax* | *S*. sp. *prionotus* | LBP 2575-15724 | **0.82** | GU210460 | GU210885 | GU210038 | GU209707 |
| Corydoradinae | Lineage 4 | *Corydoras* | *C*. cf. *hastatus* | MT104-13615 | **2.2** | GU210389 | GU210814 | GU209967 | GU209462 |
| Corydoradinae | Lineage 4 | *Corydoras* | *C. guapore* | MA73 |  | GU210364 | GU210789 | GU209943 | GU209453 |
| Corydoradinae | Lineage 4 | *Corydoras* | *C. hastatus* | LBP 1709-12815 | **2.2** | GU210405 | GU210830 | GU209983 | GU209461 |
| Corydoradinae | Lineage 4 | *Corydoras* | *C. pygmaeus* | MT51 | 2.68 | GU210498 | GU210923 | GU210076 | GU209538 |
| Corydoradinae | Lineage 5 | *Corydoras* | *C. bilineatus* | MA68 |  | GU210359 | GU210784 | GU209938 | GU209340 |
| Corydoradinae | Lineage 5 | *Corydoras* | *C. elegans* | MT23 | 2.24 | GU210468 | GU210893 | GU210046 | GU209428 |
| Corydoradinae | Lineage 5 | *Corydoras* | *C. gracilis* | MA301 |  | GU210290 | GU210715 | GU209870 | GU209451 |
| Corydoradinae | Lineage 5 | *Corydoras* | *C. nanus* | MHNG SU08-575 |  | GU210270 | GU210695 | GU209850 | GU209495 |
| Corydoradinae | Lineage 5 | *Corydoras* | *C. napoensis* | LBP 556-7227 | 1.96 | GU210410 | GU210835 | GU209988 | GU209499 |
| Corydoradinae | Lineage 5 | *Corydoras* | *C. nijsseni* | LBP 6861-32532 |  | GU210190 | GU210615 | GU209771 | GU209508 |
| Corydoradinae | Lineage 5 | *Corydoras* | *C.* sp*. ' A pauciradiatus'* | LBP 548-7187 | 1.86 | GU210430 | GU210855 | GU210008 | GU209594 |
| Corydoradinae | Lineage 5 | *Corydoras* | *C.* sp*.* 'C123 yellow cat' | MA84 |  | GU210373 | GU210798 | GU209951 | GU209608 |
| Corydoradinae | Lineage 5 | *Corydoras* | *C.* sp*.* 'C89' | MA82 |  | GU210372 | GU210797 | GU209950 | GU209637 |
| Corydoradinae | Lineage 5 | *Corydoras* | *C*. sp. 'CW18' | MA308 |  | GU210297 | GU210722 | GU209877 | GU209645 |
| Corydoradinae | Lineage 5 | *Corydoras* | *C.* sp. '*elegans* Columbia' | MA74 |  | GU210365 | GU210790 | GU209944 | GU209650 |
| Corydoradinae | Lineage 5 | *Corydoras* | *C.* sp*.* '*elegans* illuminator' | MA331 |  | GU210315 | GU210740 | GU209895 | GU209651 |
| Corydoradinae | Lineage 5 | *Corydoras* | *C. undulatus* | LBP 566-7386 | **2** | GU210441 | GU210866 | GU210019 | GU209672 |
| Corydoradinae | Lineage 6 | *Corydoras* | *C. albolineatus* | MA321 | 2.6 | GU210305 | GU210730 | GU209885 | GU209314 |
| Corydoradinae | Lineage 6 | *Corydoras* | *C.* cf*. paleatus* 'CW24' | MA61 | 4.12 | GU210354 | GU210779 | GU209933 | GU209394 |
| Corydoradinae | Lineage 6 | *Corydoras* | *C. diphyes* | MT21 |  | GU210466 | GU210891 | GU210044 | GU209420 |
| Corydoradinae | Lineage 6 | *Corydoras* | *C. ehrhardti* | LBP 741-8893 |  | GU210400 | GU210825 | GU209978 | GU209425 |
| Corydoradinae | Lineage 6 | *Corydoras* | *C. flaveolus* | MT115-12321 | **2.46** | GU210401 | GU210826 | GU209979 | GU209438 |
| Corydoradinae | Lineage 6 | *Corydoras* | *C. nattereri* | LBP 903-9697 | **1.79** | GU210411 | GU210836 | GU209989 | GU209501 |
| Corydoradinae | Lineage 6 | *Corydoras* | *C. paleatus* | LBP 567-7416 | 1.62 | GU210414 | GU210839 | GU209992 | GU209519 |
| Corydoradinae | Lineage 6 | *Corydoras* | *C. potaroensis* | MT48 |  | GU210494 | GU210919 | GU210072 | GU209624 |
| Corydoradinae | Lineage 6 | *Corydoras* | *C. reynoldsi* | MA335 |  | GU210318 | GU210743 | GU209898 | GU209545 |
| Corydoradinae | Lineage 6 | *Corydoras* | *C.* sp. 'C144' | MA86 |  | GU210374 | GU210799 | GU209952 | GU209614 |
| Corydoradinae | Lineage 6 | *Corydoras* | *C.* sp*. albolineatus* | LBP 1957-13560 |  | GU210437 | GU210862 | GU210015 | GU209592 |
| Corydoradinae | Lineage 6 | *Corydoras* | *C. tukano* | LBP 549-7195 |  | GU210440 | GU210865 | GU210018 | GU209670 |
| Corydoradinae | Lineage 7 | *Corydoras* | *C. aeneus* | MA144 | 4.4 | GU210156 | GU210581 | GU209737 | GU209310 |
| Corydoradinae | Lineage 7 | *Corydoras* | *C. eques* | MA318 |  | GU210302 | GU210727 | GU209882 | GU209436 |
| Corydoradinae | Lineage 7 | *Corydoras* | *C. melanotaenia* | MT35 | 1.64 | GU210481 | GU210906 | GU210059 | GU209486 |
| Corydoradinae | Lineage 7 | *Corydoras* | *C. rabauti* | MT54 | 2.05 | GU210501 | GU210926 | GU210079 | GU209541 |
| Corydoradinae | Lineage 7 | *Corydoras* | *C.* sp*. aeneus* 'CW10 Gold Laser' | MA42 | 2.15 | GU210340 | GU210765 | GU209919 | GU209578 |
| Corydoradinae | Lineage 7 | *Corydoras* | *C.* sp*. aeneus* 'CW9 Green Laser' | MA160 | 1.84 | GU210170 | GU210595 | GU209751 | GU209580 |
| Corydoradinae | Lineage 7 | *Corydoras* | *C.* sp*. aeneus* 'F Guyana' | MHNG 2666.037-GF03-097 | 4.12 | GU210246 | GU210671 | GU209826 | GU209576 |
| Corydoradinae | Lineage 7 | *Corydoras* | *C.* sp*. aeneus* 'Peru orange' | MA59 |  | GU210353 | GU210778 | GU209932 | GU209586 |
| Corydoradinae | Lineage 7 | *Corydoras* | *C.* sp*. aeneus* 'Peru' | LBP 1350-11447 | 3.3 | GU210432 | GU210857 | GU210010 | GU209581 |
| Corydoradinae | Lineage 7 | *Corydoras* | *C.* sp*. aeneus* 'Suriname' | MHNG 2671.014-SU05-575 |  | GU210229 | GU210654 | GU209809 | GU209587 |
| Corydoradinae | Lineage 7 | *Corydoras* | *C.* sp*. aeneus 'venezuelanus'* | MA181 | 1.79 | GU210185 | GU210610 | GU209766 | GU209673 |
| Corydoradinae | Lineage 7 | *Corydoras* | *C.* sp*. aeneus ‘macrosteus’* | MA98-27845 | 2.39 | GU210381 | GU210806 | GU209959 | GU209480 |
| Corydoradinae | Lineage 7 | *Corydoras* | *C.* sp*. aeneus ‘macrosteus’* | MA204-32760 | 1.39 | GU210207 | GU210632 | GU209787 | GU209479 |
| Corydoradinae | Lineage 7 | *Corydoras* | *C. zygatus* | MA51 |  | GU210347 | GU210772 | GU209926 | GU209683 |
| Corydoradinae | Lineage 8 | *Corydoras* | *C. ambiacus* | MA46 | 1.97 | GU210344 | GU210769 | GU209923 | GU209320 |
| Corydoradinae | Lineage 8 | *Corydoras* | *C. britski* | LBP 688-8112 | 1.67 | GU210546 | GU210971 | GU210121 | GU209298 |
| Corydoradinae | Lineage 8 | *Corydoras* | *C. multiradiatus* | MA146 |  | GU210157 | GU210582 | GU209738 | GU209299 |
| Corydoradinae | Lineage 8 | *Corydoras* | *C. splendens* | LBP 2017-14216 |  | GU210548 | GU210973 | GU210123 | GU209301 |
| Corydoradinae | Lineage 8 | *Corydoras* | *C. agassizii* | MA334 | 2.05 | GU210317 | GU210742 | GU209897 | GU209311 |
| Corydoradinae | Lineage 8 | *Corydoras* | *C.* cf*. leopardus* 'C102' | MA87 | 1.94 | GU210375 | GU210800 | GU209953 | GU209384 |
| Corydoradinae | Lineage 8 | *Corydoras* | *C. condisciplus* | MA53 |  | GU210349 | GU210774 | GU209928 | GU209405 |
| Corydoradinae | Lineage 8 | *Corydoras* | *C. crypticus* | MT17 |  | GU210461 | GU210886 | GU210039 | GU209411 |
| Corydoradinae | Lineage 8 | *Corydoras* | *C. delphax* | MT20 | 2.59 | GU210465 | GU210890 | GU210043 | GU209417 |
| Corydoradinae | Lineage 8 | *Corydoras* | *C. difluviatilis* | LBP 382-4608 | 1.94 | GU210398 | GU210823 | GU209976 | GU209418 |
| Corydoradinae | Lineage 8 | *Corydoras* | *C. ephippifer* | MA58 | 2.09 | GU210352 | GU210777 | GU209931 | GU209435 |
| Corydoradinae | Lineage 8 | *Corydoras* | *C. filamentosus* | MHNG 2707.015-SU07-625 |  | GU210240 | GU210665 | GU209820 | GU209437 |
| Corydoradinae | Lineage 8 | *Corydoras* | *C. garbei* | LBP 330-3920 |  | GU210402 | GU210827 | GU209980 | GU209442 |
| Corydoradinae | Lineage 8 | *Corydoras* | *C. geryi* | MA173 |  | GU210177 | GU210602 | GU209758 | GU209446 |
| Corydoradinae | Lineage 8 | *Corydoras* | *C. gomezi* | MT27 |  | GU210472 | GU210897 | GU210050 | GU209363 |
| Corydoradinae | Lineage 8 | *Corydoras* | *C. haraldshultzei* | MT31 | 2.42 | GU210477 | GU210902 | GU210055 | GU209458 |
| Corydoradinae | Lineage 8 | *Corydoras* | *C. imitator* | LBP 6862-32502 | 2.3 | GU210188 | GU210613 | GU209769 | GU209464 |
| Corydoradinae | Lineage 8 | *Corydoras* | *C. leopardus* | MA337 | 1.96 | GU210320 | GU210745 | GU209900 | GU209470 |
| Corydoradinae | Lineage 8 | *Corydoras* | *C. melanistius* | ANSP 180693-1460 | 2.28 | GU210330 | GU210755 | GU209910 | GU209484 |
| Corydoradinae | Lineage 8 | *Corydoras* | *C. ornatus* | MA64 |  | GU210356 | GU210781 | GU209935 | GU209513 |
| Corydoradinae | Lineage 8 | *Corydoras* | *C. pantanalensis* | LBP 691-8126 | 1.81 | GU210416 | GU210841 | GU209994 | GU209524 |
| Corydoradinae | Lineage 8 | *Corydoras* | *C. pulcher* | MT50 | 2.54 | GU210497 | GU210922 | GU210075 | GU209533 |
| Corydoradinae | Lineage 8 | *Corydoras* | *C. reticulatus* | LBP 553-7214 | 2.16 | GU210420 | GU210845 | GU209998 | GU209542 |
| Corydoradinae | Lineage 8 | *Corydoras* | *C. robinae* | MT57 | 2.27 | GU210504 | GU210929 | GU210082 | GU209548 |
| Corydoradinae | Lineage 8 | *Corydoras* | *C. robustus* | MA143 |  | GU210155 | GU210580 | GU209736 | GU209549 |
| Corydoradinae | Lineage 8 | *Corydoras* | *C. seussi* | MT60 |  | GU210508 | GU210933 | GU210086 | GU209559 |
| Corydoradinae | Lineage 8 | *Corydoras* | *C. sodalis* | LBP 530-7125 | 2.11 | GU210429 | GU210854 | GU210007 | GU209570 |
| Corydoradinae | Lineage 8 | *Corydoras* | *C.* sp*. ‘*C52’ | MA78 |  | GU210369 | GU210794 | GU209947 | GU209629 |
| Corydoradinae | Lineage 8 | *Corydoras* | *C*. sp. ‘CW13’ | MA20 |  | GU210202 | GU210627 | GU209782 | GU209641 |
| Corydoradinae | Lineage 8 | *Corydoras* | *C*. sp. 'C131 *leopardus'* | MA72 |  | GU210363 | GU210788 | GU209942 | GU209385 |
| Corydoradinae | Lineage 8 | *Corydoras* | *C.* sp. 'C141 *pulcher'* | MA175 |  | GU210179 | GU210604 | GU209760 | GU209396 |
| Corydoradinae | Lineage 8 | *Corydoras* | *C.* sp. 'C49 false *robustus'* | MT70 |  | GU210519 | GU210944 | GU210094 | GU209628 |
| Corydoradinae | Lineage 8 | *Corydoras* | *C*. sp. 'C57 nordestini' | MA184-32501 |  | GU210187 | GU210612 | GU209768 | GU209631 |
| Corydoradinae | Lineage 8 | *Corydoras* | *C*. sp. 'C66 *similis'* | MA56 |  | GU210350 | GU210775 | GU209929 | GU209632 |
| Corydoradinae | Lineage 8 | *Corydoras* | *C.* sp. 'C122 | LBP 7214-32927 |  | GU210301 | GU210726 | GU209881 | GU209634 |
| Corydoradinae | Lineage 8 | *Corydoras* | *C*. sp. 'CW6 *narcissus'* | MT73 | 2.68 | GU210522 | GU210947 | GU210097 | GU209648 |
| Corydoradinae | Lineage 8 | *Corydoras* | *C.* sp. ‘C159’ | LBP 7711-32652 |  | GU210197 | GU210622 | GU209778 | GU209655 |
| Corydoradinae | Lineage 8 | *Corydoras* | *C. spilurus* | MA76 |  | GU210367 | GU210792 | GU209946 | GU209652 |
| Corydoradinae | Lineage 8 | *Corydoras* | *C. virginae* | MT83 |  | GU210533 | GU210958 | GU210108 | GU209676 |
| Corydoradinae | Lineage 9 | *Corydoras* | *C. acrensis* | MA179 |  | GU210183 | GU210608 | GU209764 | GU209302 |
| Corydoradinae | Lineage 9 | *Corydoras* | *C. adolfoi* | LBP 6863-32527 | 3.77 | GU210189 | GU210614 | GU209770 | GU209305 |
| Corydoradinae | Lineage 9 | *Corydoras* | *C. araguaiaensis* | MA94-27706 | 4.36 | GU210377 | GU210802 | GU209955 | GU209324 |
| Corydoradinae | Lineage 9 | *Corydoras* | *C. arcuatus* | MT3 | **2.28** | GU210475 | GU210900 | GU210053 | GU209325 |
| Corydoradinae | Lineage 9 | *Corydoras* | *C. armatus* | MA111 | 4.3 | GU210136 | GU210561 | GU209718 | GU209329 |
| Corydoradinae | Lineage 9 | *Corydoras* | *C. atropersonatus* | MA303 |  | GU210292 | GU210717 | GU209872 | GU209330 |
| Corydoradinae | Lineage 9 | *Corydoras* | *C. axelrodi* | MA43 | 3.2 | GU210341 | GU210766 | GU209920 | GU209334 |
| Corydoradinae | Lineage 9 | *Corydoras* | *C. bicolor* | MHNG 2651.078-GY04-424 |  | GU210245 | GU210670 | GU209825 | GU209339 |
| Corydoradinae | Lineage 9 | *Corydoras* | *C. boesemani* | MHNG 2673.074-SU05-129 |  | GU210223 | GU210648 | GU209803 | GU209344 |
| Corydoradinae | Lineage 9 | *Corydoras* | *C. bondi* | MHNG 2651.040-GY04-123 | 2.65 | GU210244 | GU210669 | GU209824 | GU209346 |
| Corydoradinae | Lineage 9 | *Corydoras* | *C. breei* | MHNG SU08-191 |  | GU210266 | GU210691 | GU209846 | GU209349 |
| Corydoradinae | Lineage 9 | *Corydoras* | *C. brevirostris* | MT7 | 3.28 | GU210518 | GU210943 | GU210093 | GU209352 |
| Corydoradinae | Lineage 9 | *Corydoras* | *C. burgessi* | LBP 6867-32741 |  | GU210205 | GU210630 | GU209785 | GU209353 |
| Corydoradinae | Lineage 9 | *Corydoras* | *C. caudimaculatus* | LBP 562-7255 |  | GU210385 | GU210810 | GU209963 | GU209357 |
| Corydoradinae | Lineage 9 | *Corydoras* | *C.* cf*. araguaiaensis* 'C65' | MA57 | 2.64 | GU210351 | GU210776 | GU209930 | GU209365 |
| Corydoradinae | Lineage 9 | *Corydoras* | *C.* cf*. bondi* | MA165 |  | GU210172 | GU210597 | GU209753 | GU209369 |
| Corydoradinae | Lineage 9 | *Corydoras* | *C.* cf*. concolor* | LBP 2306-15843 |  | GU210387 | GU210812 | GU209965 | GU209371 |
| Corydoradinae | Lineage 9 | *Corydoras* | *C.* cf*. davidsandsi* | MA319 |  | GU210303 | GU210728 | GU209883 | GU209374 |
| Corydoradinae | Lineage 9 | *Corydoras* | *C.* cf*. guianensis* | LBP 5395-27091 |  | GU210128 | GU210553 | GU209710 | GU209382 |
| Corydoradinae | Lineage 9 | *Corydoras* | *C.* cf*. punctatus* | MA350 |  | GU210334 | GU210759 | GU209914 | GU209469 |
| Corydoradinae | Lineage 9 | *Corydoras* | *C.* cf*. sipalwini* | MHNG 2671.094-SU05-427 |  | GU210224 | GU210649 | GU209804 | GU209398 |
| Corydoradinae | Lineage 9 | *Corydoras* | *C. concolor* | MT12 |  | GU210406 | GU210831 | GU209984 | GU209402 |
| Corydoradinae | Lineage 9 | *Corydoras* | *C. copei* | MT13 |  | GU210417 | GU210842 | GU209995 | GU209575 |
| Corydoradinae | Lineage 9 | *Corydoras* | *C. coppenamensis* | MHNG 2690.017-SU01-466 |  | GU210235 | GU210660 | GU209815 | GU209407 |
| Corydoradinae | Lineage 9 | *Corydoras* | *C. cruziensis* | MA152 |  | GU210162 | GU210587 | GU209743 | GU209643 |
| Corydoradinae | Lineage 9 | *Corydoras* | *C. davidsandsi* | LBP 551-7203 | 3.57 | GU210396 | GU210821 | GU209974 | GU209414 |
| Corydoradinae | Lineage 9 | *Corydoras* | *C. duplicareus* | MA167 | 3.62 | GU210174 | GU210599 | GU209755 | GU209423 |
| Corydoradinae | Lineage 9 | *Corydoras* | *C. gossei* | MT29 |  | GU210474 | GU210899 | GU210052 | GU209448 |
| Corydoradinae | Lineage 9 | *Corydoras* | *C. griseus* | MA71 |  | GU210362 | GU210787 | GU209941 | GU209635 |
| Corydoradinae | Lineage 9 | *Corydoras* | *C. guianensis* | MHNG 2683.055-GF06-574 |  | GU210247 | GU210672 | GU209827 | GU209454 |
| Corydoradinae | Lineage 9 | *Corydoras* | *C. habrosus* | MA142 | 2.53 | GU210154 | GU210579 | GU209735 | GU209457 |
| Corydoradinae | Lineage 9 | *Corydoras* | *C. julii* | MA147 | 4.2 | GU210158 | GU210583 | GU209739 | GU209468 |
| Corydoradinae | Lineage 9 | *Corydoras* | *C. kanei* | MA349 | 3.17 | GU210332 | GU210757 | GU209912 | GU209654 |
| Corydoradinae | Lineage 9 | *Corydoras* | *C. leucomelas* | MA122 |  | GU210144 | GU210569 | GU209725 | GU209471 |
| Corydoradinae | Lineage 9 | *Corydoras* | *C. loretoensis* | MA298 | 4.3 | GU210286 | GU210711 | GU209866 | GU209474 |
| Corydoradinae | Lineage 9 | *Corydoras* | *C. loxozonus* | MA351 |  | GU210335 | GU210760 | - | GU209476 |
| Corydoradinae | Lineage 9 | *Corydoras* | *C. melini* | MA77 | 2.98 | GU210368 | GU210793 | - | GU209489 |
| Corydoradinae | Lineage 9 | *Corydoras* | *C. metae* | MT38 | 4.15 | GU210484 | GU210909 | GU210062 | GU209493 |
| Corydoradinae | Lineage 9 | *Corydoras* | *C. multimaculatus* | MA302 |  | GU210291 | GU210716 | GU209871 | GU209494 |
| Corydoradinae | Lineage 9 | *Corydoras* | *C. oiapoquensis* | MHNG 2682.023-GF06-186 | 4.5 | GU210260 | GU210685 | GU209840 | GU209511 |
| Corydoradinae | Lineage 9 | *Corydoras* | *C. osteocarus* | ANSP 185052-1477 |  | GU210323 | GU210748 | GU209903 | GU209515 |
| Corydoradinae | Lineage 9 | *Corydoras* | *C. panda* | MT41 | 2.96 | GU210487 | GU210912 | GU210065 | GU209521 |
| Corydoradinae | Lineage 9 | *Corydoras* | *C. paragua* | MA67 |  | GU210358 | GU210783 | GU209937 | GU209526 |
| Corydoradinae | Lineage 9 | *Corydoras* | *C. polystictus* | MT45 |  | GU210491 | GU210916 | GU210069 | GU209531 |
| Corydoradinae | Lineage 9 | *Corydoras* | *C. punctatus* | MHNG SU08-110 | **2.9** | GU210262 | GU210687 | GU209842 | GU209535 |
| Corydoradinae | Lineage 9 | *Corydoras* | *C. schwartzi* | LBP 1783-7120 |  | GU210421 | GU210846 | GU209999 | GU209551 |
| Corydoradinae | Lineage 9 | *Corydoras* | *C. similis* | LBP 547-7184 | 3.39 | GU210424 | GU210849 | GU210002 | GU209562 |
| Corydoradinae | Lineage 9 | *Corydoras* | *C. sipaliwini* | MHNG 2707.017-SU07-287 |  | GU210241 | GU210666 | GU209821 | GU209567 |
| Corydoradinae | Lineage 9 | *Corydoras* | *C.* sp*. 'arcuatus* super' | MT65 |  | GU210513 | GU210938 | GU210088 | GU209600 |
| Corydoradinae | Lineage 9 | *Corydoras* | *C.* sp*.* 'C121 *burgessi'* | MA178 |  | GU210182 | GU210607 | GU209763 | GU209607 |
| Corydoradinae | Lineage 9 | *Corydoras* | *C.* sp*.* 'C129' | MA149 |  | GU210159 | GU210584 | GU209740 | GU209609 |
| Corydoradinae | Lineage 9 | *Corydoras* | *C.* sp. 'C133 ornatus short snout' | MT68 |  | GU210516 | GU210941 | GU210091 | GU209617 |
| Corydoradinae | Lineage 9 | *Corydoras* | *C.* sp. 'C136' | MA305 |  | GU210294 | GU210719 | GU209874 | GU209644 |
| Corydoradinae | Lineage 9 | *Corydoras* | *C.* sp. 'C139 *oiapoquensis'* | MA174 |  | GU210178 | GU210603 | GU209759 | GU209390 |
| Corydoradinae | Lineage 9 | *Corydoras* | *C*. sp. 'C150 mazurani' | MA70 |  | GU210361 | GU210786 | GU209940 | GU209615 |
| Corydoradinae | Lineage 9 | *Corydoras* | *C.* sp. 'C30' | MA215 |  | GU210215 | GU210640 | GU209795 | GU209618 |
| Corydoradinae | Lineage 9 | *Corydoras* | *C.* sp. 'C43' | MA80 | 4.8 | GU210371 | GU210796 | GU209949 | GU209626 |
| Corydoradinae | Lineage 9 | *Corydoras* | *C.* sp. 'C91 Peru *bondi'* | MA300 | 2.78 | GU210289 | GU210714 | GU209869 | GU209638 |
| Corydoradinae | Lineage 9 | *Corydoras* | *C.* sp. 'CW21 *axelrodi'* | MA320 |  | GU210304 | GU210729 | GU209884 | GU209642 |
| Corydoradinae | Lineage 9 | *Corydoras* | *C.* sp*. arcuatus* 'Rio Negro' | LBP 7709-32609 |  | GU210196 | GU210621 | GU209777 | GU209599 |
| Corydoradinae | Lineage 9 | *Corydoras* | *C.* sp*. armatus* 'Green cana' | MA330 |  | GU210314 | GU210739 | GU209894 | GU209602 |
| Corydoradinae | Lineage 9 | *Corydoras* | *C.* sp*. breei* | MHNG SU08-583 |  | GU210278 | GU210703 | GU209858 | GU209603 |
| Corydoradinae | Lineage 9 | *Corydoras* | *C.* sp*.* ‘C84’ | MA197-32671 |  | GU210199 | GU210624 | - | GU209490 |
| Corydoradinae | Lineage 9 | *Corydoras* | *C.* sp. ’CW28’ | MA110 |  | GU210135 | GU210560 | GU209717 | GU209612 |
| Corydoradinae | Lineage 9 | *Corydoras* | *C.* sp*. davidsandsi* | MA134 |  | GU210152 | GU210577 | GU209733 | GU209649 |
| Corydoradinae | Lineage 9 | *Corydoras* | *C.* sp*. melini* | MA333 |  | GU210316 | GU210741 | GU209896 | GU209636 |
| Corydoradinae | Lineage 9 | *Corydoras* | *C. sterbai* | MT75 | 3.16 | GU210524 | GU210949 | GU210099 | GU209662 |
| Corydoradinae | Lineage 9 | *Corydoras* | *C. trilineatus* | MT80 | 3.4 | GU210530 | GU210955 | GU210105 | GU209667 |
| Corydoradinae | Lineage 9 | *Corydoras* | *C. weitzmani* | MA35 | 2.56 | GU210333 | GU210758 | GU209913 | GU209680 |
| Outgroups |  |  |  |  |  |  |  |  |  |
|  |  |  | *Species* |  |  |  |  |  |  |
| Claridae |  |  | *Clarias batrachus* |  |  | KC572134.1 | KC572134.1 | KC572134.1 | KC572134.1 |
| Claridae |  |  | *Heteropneustes fossilis* |  |  | KP345880.1 | KP345880.1 | KP345880.1 | KP345880.1 |
| Ictaluridae |  |  | *Cranoglanis bouderius* |  |  | AY898626.1 | AY898626.1 | AY898626.1 | AY898626.1 |
| Ictaluridae |  |  | *Ictalurus punctatus* |  |  | AF482987.1 | AF482987.1 | AF482987.1 | AF482987.1 |
| Arioidea |  |  | *Galeichthys feliceps* |  |  | FJ013178.1 | FJ013178.1 | - | FJ013162.1 |
| Arioidea |  |  | *Gogo arcuatus* |  |  | FJ013176.1 | FJ013191.1 | - | FJ013160.1 |
| Characiformes |  |  | *Pygocentrus nattereri* |  |  | AP012000.1 | AP012000.1 | AP012000.1 | AP012000.1 |
| Characiformes |  |  | *Hemiodus_gracilis.* |  |  | AP011990.1 | AP011990.1 | AP011990.1 | AP011990.1 |
| Gonorynchiformes |  |  | *Chanos chanos* |  |  | AB054133.1 | AB054133.1 | AB054133.1 | AB054133.1 |
| Gonorynchiformes |  |  | *Gonorynchus abbreviatus* |  |  | AP009402.1 | AP009402.1 | AP009402.1 | AP009402.1 |
| Cyprinidae |  |  | *Cyprinus carpio* |  |  | X61010.1 | X61010.1 | X61010.1 | X61010.1 |
| Cyprinidae |  |  | *Danio margaritatus* |  |  | AP011404.1 | AP011404.1 | AP011404.1 | AP011404.1 |
| Cyprinidae |  |  | *Cobitis striata* |  |  | AP010782.1 | AP010782.1 | AP010782.1 | AP010782.1 |
| Cyprinidae |  |  | *Moxostoma poecilurum* |  |  | AB242167.1 | AB242167.1 | AB242167.1 | AB242167.1 |
| Clupeiformes |  |  | *Engraulis encrasicolus* |  |  | AP009137.1 | AP009137.1 | AP009137.1 | AP009137.1 |
|  |  |  |  |  |  |  |  |  |  |

Genome size estimates in bold taken from [www.genomesize.com](http://www.genomesize.com).

*value from *A. fuscoguttatus* assigned to A. cf poecilius as only genome size available for this lineage

**Supplementary Table 2. Fossil calibrations used and their sources.**

1. tMRCA Callichthyidae. Corydoras revelatus fossil dated at approximately 58.2-58.5 million years bp.

Source Reis [1] Prior setting: Lognormal distribution offset=55, Mean =1.4 S=0.94. 95% upper bound 74 mya. Calibration follows Santini et al. [2].

2. tMRCA of the clade (Clarias, Heteropneustes): First appearance of the African Clariidae (34–56 Ma). Source Gayet et al [3]. Prior setting: Lognormal distribution, offset = 34, M=1.0, S=1.25. 95% upper bound 55.2 mya.

3. tMRCA of the clade (Ictalurus, Cranoglanis): The oldest ictalurid stem fossil Astephus sp. Source Lundberg [4] from the Polecat Bench Formation, Wyoming from Early Paleocene (63–65 Ma). Prior setting: offset = 63 Mya, M=1.0, S=1.25.

4. tMRCA of the clade Arioidea (Gogo, Ariopsis). Calibration source: Broughton et al. [5]. Prior setting: Lognormal distribution, offset 63, M=1.0, S=1.25. 95% upper bound 84.2 mya.

5. tMRCA Serrasalmidae + Hemiodontidae. (Pygocentrus, Hemiodus). Pacu teeth – oldest serrasalmid

fossils [6]. Calibration source: Broughton et al. [5]. Lognormal priors, offset= 61.0 Ma. M=1.0, S=1.25. 95% upper bound 82.2 mya.

6. tMRCA of the clade (Danio, Cyprinus): The †Parabarbus fossil (Cyprinidae) from the Ypresian of the

Obailinskaya Formation, Zaissan Basin, Kazakhstan (51–49 Ma) is the oldest crown cyprinid. Source Saito et al [7]. Lognormal distribution offset = 49mya, M=1, S=1.25. 95% upper bound 77.2 mya.

7. tMRCA of the clade Gonorynchyformes (Chanos, Gonorynchus forsteri) Hard minimum age: 139.0 Ma. Calibration source: Broughton et al. [5]. Prior setting: Lognormal distribution, offset = 133, M=1.0, S=1.25. 95% upper bound 154 mya.

**Calibration sources:**

1. Reis RE. In: Malabarba L.R. Vari R.P. LZMS, and Lucena C.A.S., Reis R E, editor. Systematics, Biogeography, and the Fossil Record of the Callichthyidae: A Review of the Available Data

Phylogeny and Classification of Neotropical Fishes. Porto Allegre, Brasil: EDIPUCRS; 1998. p. 603.

2. Santini F, Harmon LJ, Carnevale G, Alfaro ME (2009) Did genome duplication drive the origin of teleosts? A comparative study of diversification in ray-finned fishes. BMC Evol Biol 9:194.

3. Gayet M, Meunier FJ. Paleontology and palaeobiogeography of catfishes

In: Arratia G, Kapoor BG, Chardon M, Diogo R, editors. Catfishes. Volume 2 Enfield,

NH: Science Publishers; 2003. pp. 491–522.

4. Lundberg JG. The fossil catfishes of North America. Univ Mich Mus Paleont Pap Paleont. 1975;11: 1–51.

5. Broughton RE, Betancur-R. R, Li C, Arratia G, Orti G (2013) Multi-locus phylogenetic analysis reveals

the pattern and tempo of bony fish evolution. PLOS Currents Tree of Life.

6. Gayet M., Meunier F.J. 1998 Maastrichtian to early Late Paleocene Freshwater osteichthyes of Bolivia:

Additions and comments. In Phylogeny and Classification of Neotropical Fishes

(eds. Malabarba L.R., Reis R.E., Vari R.P., Lucena Z.M.S., Lucena C.A.S.), pp. 85-110.

Porto Alegre, EDIPUCRS.

7. Saitoh K, Sado T, Doosey MH, Bart Jr HL, Inoue JG, et al. (2011) Evidence from mitochondrial genomics supports the lower Mesozoic of South Asia as the time and place of basal divergence of cypriniform fishes (Actinopterygii: Ostariophysi). Zoological Journal of the Linnean Society 161: 633-662.

Supplementary Table 3. Percentage of bases identified in each individual as one of five TE

|  | **Retroelements** | | | **DNA Transposons** | |  |
| --- | --- | --- | --- | --- | --- | --- |
| **Species** | **SINEs** | **LINEs** | **LTR elements** | **hobo-Activator** | **TC1-IS630-Pogo** | **Total masked** |
| *Megalechis sp.* | 1.81 | 1.62 | 0.35 | 0.26 | 20.68 | 42.07 |
| *C. fowleri 1* | 0.91 | 3.26 | 1.78 | 0.2 | 0.62 | 10.5 |
| *C. fowleri 2* | 0.94 | 3.19 | 1.61 | 0.21 | 0.88 | 10.49 |
| *A. poecilius 1* | 1.43 | 1.45 | 1.24 | 0.22 | 3.49 | 11.66 |
| *A. poecilius 2* | 1.28 | 1.37 | 1.13 | 0.22 | 3.23 | 10.96 |
| *S. kronei 1* | 0.84 | 1.94 | 1.29 | 0.24 | 4.91 | 12.33 |
| *S. kronei 2* | 0.92 | 1.92 | 1.38 | 0.29 | 4.94 | 12.9 |
| *C. pygmaeus 1* | 2.03 | 4.04 | 2.59 | 0.47 | 5.53 | 18.36 |
| *C. pygmaeus 2* | 4.15 | 3.98 | 5.5 | 0.55 | 6.12 | 25.98 |
| *C. elegans 1* | 0.96 | 4.62 | 1.68 | 0.36 | 9.85 | 21.45 |
| *C. elegans 2* | 0.96 | 4.42 | 1.7 | 0.36 | 8.92 | 20.23 |
| *C. nattereri 1* | 0.8 | 2.25 | 5.62 | 1.54 | 6.83 | 20.34 |
| *C. nattereri 2* | 0.92 | 2.38 | 5.37 | 1.37 | 6.72 | 20.52 |
| *C. aeneus 1* | 0.42 | 0.7 | 1.26 | 0.38 | 48.2 | 52.59 |
| *C. aeneus 2* | 0.47 | 0.73 | 1.41 | 0.59 | 45.05 | 50.28 |
| *C. imitator 1* | 1.64 | 2.53 | 2.16 | 1.22 | 22.46 | 33.58 |
| *C. imitator 2* | 2.04 | 2.55 | 2.64 | 1.23 | 21.45 | 33.86 |
| *C. metae 1* | 0.39 | 0.60 | 2.72 | 0.64 | 70.22 | 74.85 |
| *C. metae 2* | 0.47 | 0.94 | 3.42 | 1.75 | 60.27 | 67.06 |
| *C. araguaiaensis 1* | 0.59 | 1.19 | 8.19 | 1.73 | 36.78 | 48.75 |
| *C. araguaiaensis 2* | 0.56 | 1.18 | 8.26 | 2.18 | 35.22 | 47.66 |

Supplementary Table 4. RAD sequence data assembly statistics for each species, read numbers per replicate.

| **Lineage** | **Sample** | **#Reads (millions)** | **Velvet-Contigs** | **N50** | **Mean Depth** | **Contigs after filtering** |
| --- | --- | --- | --- | --- | --- | --- |
| 0 | *Megalechis-*Rep*1 sp.* | 3.35 | 37,345 | 432 | 45.26 | **36,426** |
| 0 | *Megalechis* Rep2 | 3.00 |  |  |  |  |
| 1 | Fow1-Rep1 | 2.36 | 28,047 | 414 | 40.39 | **26,743** |
| 1 | Fow1-Rep2 | 2.69 |  |  |  |  |
| 1 | Fow2-Rep1 | 2.71 |  |  |  |  |
| 1 | Fow2-Rep2 | 2.54 |  |  |  |  |
| 2 | Apo1-Rep1 | 1.38 | 20,081 | 299 | 97.675 | **18,374** |
| 2 | Apo1-Rep2 | 5.24 |  |  |  |  |
| 2 | Apo2-Rep1 | 5.15 |  |  |  |  |
| 2 | Apo2-Rep2 | 7.25 |  |  |  |  |
| 3 | Skro1-Rep1 | 2.25 | 24,100 | 335 | 41.025 | **20,866** |
| 3 | Skro1-Rep2 | 4.03 |  |  |  |  |
| 3 | Skro2-Rep1 | 3.06 |  |  |  |  |
| 3 | Skro2-Rep2 | 3.06 |  |  |  |  |
| 4 | Pyg1-Rep1 | 11.51 | 34,490 | 355 | 116.18 | **30,051** |
| 4 | Pyg1-Rep2 | 11.28 |  |  |  |  |
| 4 | Pyg2-Rep1 | 12.15 |  |  |  |  |
| 4 | Pyg2-Rep2 | 11.15 |  |  |  |  |
| 5 | Eleg1-Rep1 | 8.61 | 22,238 | 290 | 49.63 | **18,674** |
| 5 | Eleg1-Rep2 | 6.50 |  |  |  |  |
| 5 | Eleg2-Rep1 | 8.77 |  |  |  |  |
| 5 | Eleg2-Rep2 | 5.77 |  |  |  |  |
| 6 | Nat1-Rep1 | 3.75 | 13,166 | 270 | 49.625 | **11,972** |
| 6 | Nat1-Rep2 | 5.37 |  |  |  |  |
| 6 | Nat2-Rep1 | 6.11 |  |  |  |  |
| 6 | Nat2-Rep2 | 4.57 |  |  |  |  |
| 7 | Aen1-Rep1 | 7.99 | 19,375 | 292 | 55.875 | **17,787** |
| 7 | Aen1-Rep2 | 2.85 |  |  |  |  |
| 7 | Aen2-Rep1 | 5.25 |  |  |  |  |
| 7 | Aen2-Rep2 | 6.62 |  |  |  |  |
| 8 | Imi1-Rep1 | 7.58 | 58,604 | 447 | 54.825 | **49,552** |
| 8 | Imi1-Rep2 | 8.03 |  |  |  |  |
| 8 | Imi2-Rep1 | 8.72 |  |  |  |  |
| 8 | Imi2-Rep2 | 9.13 |  |  |  |  |
| 9 | Me1-Rep1 | 11.19 | 44,052 | 397 | 104.175 | **40,763** |
| 9 | Me1-Rep2 | 13.06 |  |  |  |  |
| 9 | Me2-Rep1 | 20.10 |  |  |  |  |
| 9 | Me2-Rep2 | 16.33 |  |  |  |  |
| 9 | Ara1-Rep1 | 15.46 | 42,224 | 403 | 145.865 | **40,026** |
| 9 | Ara1-Rep2 | 13.19 |  |  |  |  |
| 9 | Ara2-Rep1 | 13.06 |  |  |  |  |
| 9 | Ara2-Rep2 | 15.62 |  |  |  |  |

Supplementary Table 5: Details of samples and barcodes used in RAD library sequencing

| **Species** | **Indiv/Replicate** | **P1 Barcode** | **P2 Barcodes** | **RAD Library** | **Seq Run** |
| --- | --- | --- | --- | --- | --- |
|  |  |  |  |  |  |
| *Megalechis sp.* | 1/1 | TCAGA | AGTCA & TACGTC | 1 |  |
|  | 1/2 | CGTATCA | AGTCA & TACGTC |  |  |
|  |  |  |  |  |  |
| *C. fowleri* | 1/1 | GATCG | AGTCA & TACGTC | 2 |  |
|  | 1/2 | TGCAACA | AGTCA & TACGTC |  |  |
|  | 2/1 | AGAGT | AGTCA & TACGTC |  |  |
|  | 2/2 | TCTCTCA | AGTCA & TACGTC |  |  |
|  |  |  |  |  |  |
| *C. imitator* | 1/1 | ATCGA | AGTCA & TACGTC | 3 |  |
|  | 1/2 | CACAGAC | AGTCA & TACGTC |  |  |
|  | 2/1 | TCGAG | AGTCA & TACGTC |  | 1 |
|  | 2/2 | GTACACA | AGTCA & TACGTC |  |  |
|  |  |  |  |  |  |
| *C. metae* | 1/1 | GCATT | AGTCA & TACGTC | 4 |  |
|  | 1/2 | CAGTCAC | AGTCA & TACGTC |  |  |
|  | 2/1 | ATGCT | AGTCA & TACGTC |  |  |
|  | 2/2 | CAGTCCA | AGTCA & TACGTC |  |  |
|  |  |  |  |  |  |
| *C. araguaiaensis* | 1/1 | ACGTA | AGTCA & TACGTC | 5 |  |
|  | 1/2 | CTCTTCA | AGTCA & TACGTC |  |  |
|  | 2/1 | GACTA | AGTCA & TACGTC |  |  |
|  | 2/2 | AGGACAC | AGTCA & TACGTC |  |  |
|  |  |  |  |  |  |
| *A. poecilius* | 1/1 | TCAGA | AGTCA | 6 |  |
|  | 2/1 | TGCAACA | AGTCA |  |  |
|  | 1/2 | GATCG | AGTCA |  |  |
|  | 2/2 | CGTATCA | AGTCA |  |  |
|  |  |  |  |  |  |
| *S. kronei* | 1/1 | ATCGA | TACGTC | 7 |  |
|  | 2/1 | ACTGCAC | TACGTC |  |  |
|  | 1/2 | TCGAG | TACGTC |  |  |
|  | 2/2 | TCTCTCA | TACGTC |  |  |
|  |  |  |  |  |  |
| *C. pygmaeus* | 1/1 | GTCAC | AGTCA | 8 |  |
|  | 2/1 | GTACACA | AGTCA |  |  |
|  | 1/2 | GCATT | AGTCA |  | 2 |
|  | 2/2 | CTCTTCA | AGTCA |  |  |
|  |  |  |  |  |  |
| *C. elegans* | 1/1 | ACGTA | TACGTC | 9 |  |
|  | 2/1 | CTAGGAC | TACGTC |  |  |
|  | 1/2 | AGAGT | TACGTC |  |  |
|  | 2/2 | CAGTCAC | TACGTC |  |  |
|  |  |  |  |  |  |
| *C. nattereri* | 1/1 | ATGCT | AGTCA | 10 |  |
|  | 2/1 | GCTAACA | AGTCA |  |  |
|  | 1/2 | GACTA | AGTCA |  |  |
|  | 2/2 | AGGACAC | AGTCA |  |  |
|  |  |  |  |  |  |
| *C. aeneus* | 1/1 | GATCG | TACGTC | 11 |  |
|  | 2/1 | CTCTTCA | TACGTC |  |  |
|  | 1/2 | GCATT | TACGTC |  |  |
|  | 2/2 | AGGACAC | TACGTC |  |  |

Supplementary Table 6. Number of contigs with one, two or multiple haplotypes as identified by Hapler.

|  |  |  | Haplotype Number per contig | | | |
| --- | --- | --- | --- | --- | --- | --- |
| Lineage | Sample | Genome size | One | Two | Multicopy | Total |
| 0 | Megalechis | 0.85 | 7026 | 1217 | 423 | 8666 |
| 1 | *C.fowleri 1* | 0.65 | 4480 | 788 | 384 | 5653 |
| 1 | *C.fowleri2* |  | 3280 | 851 | 399 | 4531 |
| 2 | *A.c.f.poecilius1* | 0.76* | 1790 | 1127 | 846 | 3765 |
| 2 | *A.c.f.poecilius2* |  | 1555 | 1202 | 981 | 3740 |
| 3 | *S.kronei1* | 0.94 | 1995 | 1388 | 840 | 4226 |
| 3 | *S.kronei2* |  | 1920 | 1545 | 1007 | 4475 |
| 4 | *C.pygmaeus 1* | 2.68 | 1314 | 849 | 851 | 3018 |
| 4 | *C.pygmaeus2* |  | 1338 | 689 | 797 | 2828 |
| 5 | *C. elegans1* | 2.24 | 2378 | 1676 | 978 | 5037 |
| 5 | *C.elegans 2* |  | 2482 | 1662 | 936 | 5085 |
| 6 | *C.nattereri1* | 1.79 | 2020 | 1217 | 626 | 3869 |
| 6 | *C.nattereri2* |  | 2024 | 1292 | 647 | 3969 |
| 7 | *C.aenus1* | 4.4 | 2141 | 1285 | 764 | 4197 |
| 7 | *C.aenus2* |  | 2389 | 1136 | 677 | 4209 |
| 8 | *C.imitator1* | 2.3 | 4640 | 1286 | 1152 | 7086 |
| 8 | *C.imitator2* |  | 4201 | 1225 | 1030 | 6464 |
| 9 | *C. metae 1* | 4.15 | 632 | 862 | 1436 | 2939 |
| 9 | *C.metae2* |  | 912 | 1088 | 1698 | 3707 |
| 9 | *C.araguaiaensis1* | 4.36 | 1274 | 1236 | 1762 | 4281 |
| 9 | *C.araguaiaensis2* |  | 1272 | 1291 | 1990 | 4562 |

* value from *A. fuscoguttatus* assigned to A. cf poecilius as this is the only genome size available for this lineage
